# Decaborane: From Alfred Stock and Rocket Fuel Projects to Nowadays †

**DOI:** 10.3390/molecules28176287

**Published:** 2023-08-28

**Authors:** Igor B. Sivaev

**Affiliations:** A.N. Nesmeyanov Institute of Organoelement Compounds, Russian Academy of Sciences, 28 Vavilov Str., 119334 Moscow, Russia; sivaev@ineos.ac.ru

**Keywords:** decaborane, history, properties, derivatives

## Abstract

The review covers more than a century of decaborane chemistry from the first synthesis by Alfred Stock to the present day. The main attention is paid to the reactions of the substitution of hydrogen atoms by various atoms and groups with the formation of *exo*-polyhedral boron–halogen, boron–oxygen, boron–sulfur, boron–nitrogen, boron–phosphorus, and boron–carbon bonds. Particular attention is paid to the chemistry of *conjucto*-borane *anti*-[B_18_H_22_], whose structure is formed by two decaborane moieties with a common edge, the chemistry of which has been intensively developed in the last decade.

## 1. Introduction

Decaborane [B_10_H_14_] plays a central role in the chemistry of polyhedral boron hydrides. Decaborane is an essential boron reagent for the preparation of medium and higher carboranes C_2_B_n_H_n+2_ (n = 8–10) [1] and the carba-*closo*-decaborate anions [CB_9_H_10_]^−^ [2]. Until recently, the synthesis of the *closo*-dodecaborate [B_12_H_12_]^2−^ [3,4] and the carba-*closo*-dodecaborate [CB_11_H_12_]^−^ [5,6] anions was also based on the use of decaborane, and it is still used for the synthesis of the *closo*-decaborate anion [B_10_H_10_]^2−^ [7,8]. In addition, decaborane can be used to prepare boron coatings [9,10,11,12,13], nanoparticles [14], microcrystals [15,16], boron nitride nanosheets [17], and nanotubes [18], as well as various metal boride thin films [19,20,21,22,23,24]. Recently, a decaborane-based fuel cell power source with a high energy density was developed [25]. The intensive development of the chemistry of decaborane is associated with the 1950s to the early 1960s, when the main types of its transformations were discovered and described. These early studies were reviewed in the 1960s by Hawthorne [26] and Zakharkin et al. [27]. This area was also partly elucidated in *Boron Hydride Chemistry* [28] and *Comprehensive Inorganic Chemistry I* [29]. Recent studies in the field of decaborane chemistry, deepening and expanding the previously described conclusions using modern instrumental methods, were briefly covered in *Comprehensive Inorganic Chemistry III* [30]. Therefore, the purpose of this review is to give the most complete picture of the current state of the chemistry of decaborane and its derivatives.

## 2. Synthesis, Structure, and General Properties

The formation of this ten-vertex cluster during the pyrolysis of diborane B_2_H_6_ was first described by Alfred Stock and co-workers more than 100 years ago [31,32]. The best yields of decaborane(14) were obtained by heating diborane to 120 °C for 47 h. The low volatility of decaborane allows it to be easily separated from other volatile boron hydrides while being volatile enough to be easily separated from non-volatile products. Decaborane is a colorless, air-stable, easily subliming, malodorous, crystalline solid that melts at 99.7 °C and boils with decomposition at 213 °C [33]. For a long time, interest in the chemistry of boron hydrides was mainly academic but was supported by the fact that boranes and some related compounds did not comply with the usual rules relating the chemical composition to the classical theory of valence. At the same time, various assumptions were made about the structure of B_10_H_14_, including linear [34] or naphthalene-like [35] structures.

**Figure 1:**
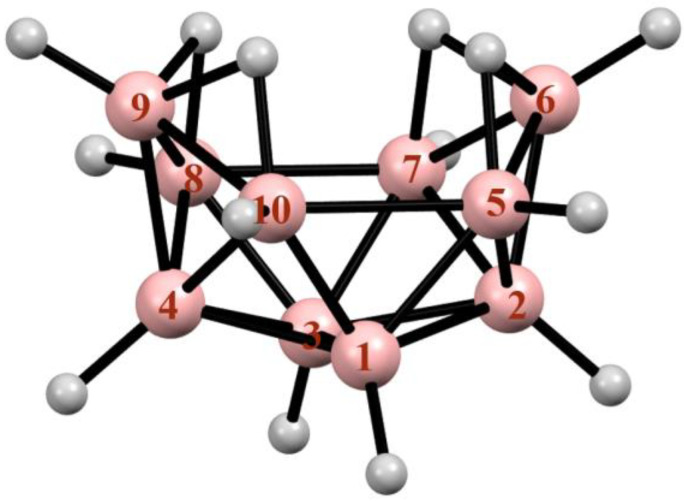
Structure and numbering of atoms in decaborane B_10_H_14_.

Practical interest in boron hydrides, and decaborane in particular, arose shortly after World War II, when the United States government launched programs (Projects Hermes, Zip, and HEF (High-Energy Fuels)) [36,37] whose purpose was to develop borane-based aviation and rocket fuels capable of generating much higher energy than conventional kerosene-based fuels [38,39,40]. As a part of this program, two chemical companies, Callery Chemical Company and Olin-Mathieson Corporation, developed eight pilot and production plants and produced an array of borane-derived energetic products, including methyldecaborane (HEF-4), ethyldecaborane (HEF-3), and ethylacetylenedecaborane (HEF-5), to be tested as additives to propellants and explosives [41]. Amost at the same time, due to the development of various physical research methods, such as single-crystal X-ray diffraction, neutron diffraction, and gas phase electron diffraction, the molecular structure of [B_10_H_14_] was determined [42,43,44,45,46,47,48,49,50]. The decaborane molecule was found to be shaped like a boat built from ten BH-units, with four additional BHB bridges decorating its bow and stern (Figure 1).

Shortly thereafter, the future Nobel Winner Lipscomb and his collaborators developed bond counting rules and topological principles that made it possible to describe bonding in boron hydrides. According to the topological formalism, the binding in the decaborane molecule can be described by a combination of four 3c-2e B-H-B bonds, six closed or fractional closed 3c-2e B-B-B bonds, and two 2c-2e B-B bonds [51,52,53]. Some time later, molecular orbital theory in the form of the extended Hückel theory was originated and applied to decaborane to provide an alternative to this topological approach [54,55,56]. Subsequently, both the logical basis and the parameters for these molecular orbitals were greatly improved using the more rigorous molecular self-consistent field (SCF) method [57,58]. More recently, the electronic structure of decaborane has been described in terms of the BadeR′s theory “Atoms in Molecules” (AIM) [59].

A powerful tool for determining the structure of polyhedral boron hydrides is NMR spectroscopy, the practical birth of which coincided with a wave of interest in the chemistry of boron hydrides. Therefore, it is not surprising that decaborane was one of the first molecules to be investigated using NMR spectroscopy [60,61,62]. The subsequent development of the instrumental base and methods of NMR spectroscopy caused repeated studies [63,64,65,66,67,68,69,70]. The decaborane molecule has also been characterized by IR [59,71,72], Raman [59], electron [73,74,75], NQR [76,77], photoelectron [78], and electron energy loss [79] spectroscopy. The ionization potentials of decaborane and ^11^B-enriched decaborane were determined to be 11.0 eV [80] and 10.26 eV [81], respectively. The dipole moment of decaborane was determined by measuring the dielectric constants of benzene, cyclohexane, and carbon disulfide solutions and varies from 3.17 D in carbon disulfide to 3.62 D in benzene [82]. The magnetic susceptibility of decaborane is −116 ± 1.5 × 10^−6^ emu mol^−1^ [82]. The heat of formation of decaborane was determined to be −66.1 kJ/mol [83]. The heat capacity of decaborane has been measured, and the derived thermodynamic functions have been calculated [84,85]. The heats of melting and vaporization [85], as well as the vapor pressure of decaborane [85,86], were also determined. Pressure-induced room temperature transformations of decaborane up to 131 GPa were studied using in situ optical spectroscopy techniques [87].

The industrial production of boron hydrides, which involved more than 2000 people, was accompanied by various accidents, which led to the discovery of the high toxicity of decaborane and its derivatives [88,89,90,91,92]. With decaborane, intoxication, headaches, tremors, impaired coordination, confusion, anxiety, photophobia, and other symptoms are observed. Moreover, intoxication can occur from relatively small amounts of decaborane. Decaborane can be detected by its odor at or near its maximum acceptable concentration, but there is considerable olfactory fatigue. Repeated exposure to decaborane can cause severe damage to the nervous system [93]. The effects of decaborane on various animals have also been studied [93,94,95,96,97,98,99,100,101,102,103,104,105,106,107,108,109,110,111]. A number of studies were directed to study the mechanism of decaborane action on living organisms [112,113,114,115,116,117,118,119,120,121,122,123,124,125,126,127,128,129,130].

**Scheme 1:**
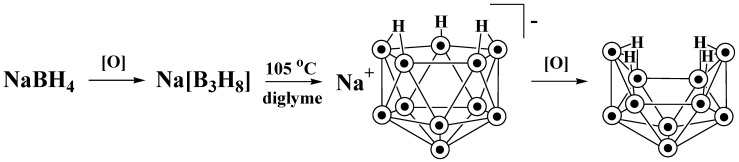
Synthesis of decaborane(14) from sodium tetrahydroborate NaBH_4_.

The production of decaborane, established in the 1950s, was based on the pyrolytic conversion of diborane proposed by Alfred Stock [33]. At the same time, attempts were made to find an alternative to this dangerous process, among which the use of the CW CO_2_ laser is worth mentioning [131]. Almost at the same time, a convenient and effective method was proposed, which is based on the oxidation of sodium tetrahydroborate NaBH_4_ to the octahydrotriborate anion [B_3_H_8_]^−^, followed by its pyrolysis in diglyme at 105 °C to the tetradecahydro-*nido*-undecaborate anion [B_11_H_14_]^−^ [132]. The subsequent mild oxidation of [B_11_H_14_]^−^ gives decaborane [B_10_H_14_] (Scheme 1) [133,134,135,136]. Decaborane can also be obtained by the cage-opening of the *closo*-decaborate anion [B_10_H_10_]^2−^ on protonation with strong acids such as sulfuric acid [137].

**Scheme 2:**
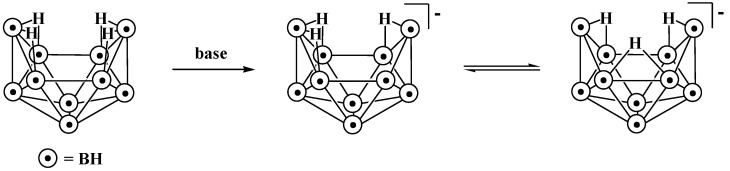
Preparation of the [B_10_H_13_]^−^ anion and its tautomeric forms.

Decaborane(14) has an acidic character [138] and can be deprotonated with strong bases, such as sodium hydride [139], tetraalkylammonium hydroxides [140], diethylamine [140], triethylamine [140,141], methylenetriphenylphosphorane [141,142], or a Proton Sponge (PS) [143,144] to give the corresponding salts of the tridecahydro-*nido*-decaborate [B_10_H_13_]^−^ anion (Scheme 2). The p*K*_a_ value of decaborane(14) in aqueous ethanol was found to vary from 2.41 to 3.21 depending on the water content [145].

The (Et_3_NH)[B_10_H_13_] and (Et_4_N)[B_10_H_13_] salts obtained by the deprotonation of decaborane with Et_3_N and (Et_4_N)OH, respectively, have been found to trigger the hypergolic reactivity of some polar aprotic organic solvents, such as tetrahydrofuran and ethyl acetate [146].

**Figure 2:**
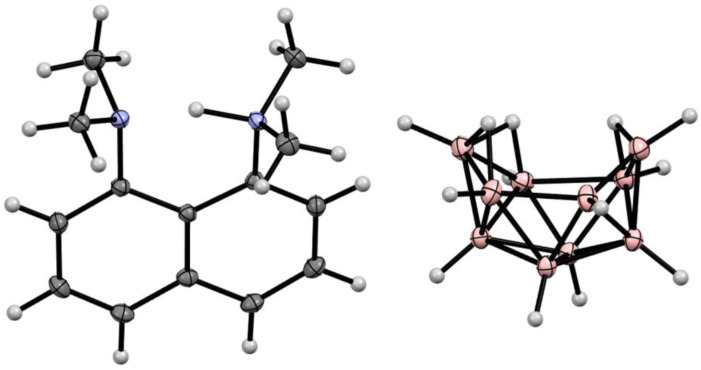
Solid state structures of the (HPS)^+^ cation (**left**) and of the [*nido*-B_10_H_13_]^−^ anion (**right**) in the crystal structure of (HPS)[B_10_H_13_].

The solid state structures of (Et_3_NH)[B_10_H_13_] [147], (BnNMe_3_)[B_10_H_13_] [148], and (HPS)[B_10_H_13_] [144] were determined by single-crystal X-ray diffraction. The solid state structure of the [B_10_H_13_]^−^ anion (Figure 2) can be derived from the structure of B_10_H_14_ by μ-H(9,10) deprotonation.

In the solution, the [B_10_H_13_]^−^ anion exists as a mixture of symmetrical and unsymmetrical *H*-tautomers with different arrangements of bridging hydrogens (Figure 2) [143,149], with an interconversion ΔG^‡^ value of less than 7 kcal/mol [144].

Strong bases such as sodium hydride in ether solvents are able to remove two protons from decaborane(14) to form the [B_10_H_12_]^2−^ dianion [140,150,151]. The latter is unstable in solution and transforms into other decaborates and their derivatives [151]. According to quantum chemical calculations, the [B_10_H_12_]^2−^ anion has the *C*_2_-symmetric structure with μ-B(5)HB(6) and μ-B(8)HB(9) bridging hydrogens [152].

**Scheme 3:**
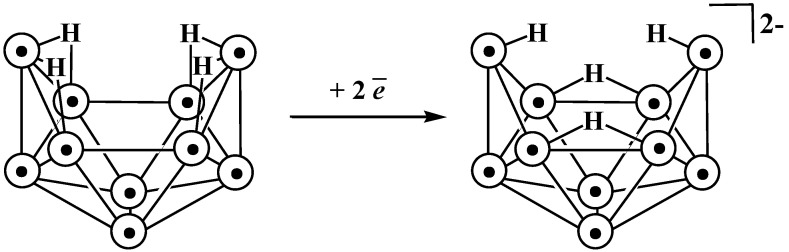
Preparation of the [*arachno*-B_10_H_14_]^2−^ anion.

The reduction of decaborane(14) with KBH_4_ in water results in the formation of the [*arachno*-B_10_H_14_]^2−^ anion with a boron cage geometry near the same as that of the starting [*nido*-B_10_H_14_] (Scheme 3), which was isolated by precipitation from an aqueous solution in the form of rubidium, cesium, or tetramethylammonium salts [153,154]. The structure of the [B_10_H_14_]^2−^ anion was proposed using ^11^B NMR spectroscopy [155]. The solid state structure of (Me_4_N)_2_[B_10_H_14_] was determined by single-crystal X-ray diffraction [154]. It was supposed that the reaction proceeds by hydride transfer with the formation of the [B_10_H_15_]^−^ anion. The latter is unstable in the solution and loses hydrogen to form [*nido*-B_10_H_13_]^−^ [156,157]. 

It should be borne in mind that decaborane itself has pronounced reducing properties. Due to this, the possibility of its use as a reducing agent in organic synthesis was studied. In particular, decaborane can be used for the reduction of acetals to ethers [158], the reductive esterification of aromatic aldehydes [159,160], and the reductive amination of acetals with aromatic amines [161]. Decaborane can also be used for chemoselective reduction aldehydes and ketones [162,163,164], the dehalogenation of α-halocarbonyl compounds [165], and the hydrogenation of alkenes or alkynes [166]. 

The bridging hydrogens in decaborane(14) were found to exchange rapidly for deuterium atoms with D_2_O in 1,4-dioxane or acetonitrile to give [μ_4_-B_10_H_10_D_4_] [72,167,168]. The use of DCl in 1,4-dioxane makes it possible to obtain the decadeuterated decaborane [μ_4_-5,6,7,8,9,10-B_10_H_4_D_10_] [169]. The treatment of decaborane(14) with DCl in a carbon disulfide solution and in the presence of AlCl_3_ results in the tetradeuterated decaborane [1,2,3,4-B_10_H_10_D_4_] [169,170]. If the reaction is carried out under heating in sealed ampoule, the product is the octadeuterated decaborane [1,2,3,4,5,7,8,10-B_10_H_6_D_8_] [72]. The tetradeuterated decaborane [5,7,8,10-B_10_H_10_D_4_] was obtained by the reaction of [1,2,3,4,5,7,8,10-B_10_H_6_D_8_] with HCl in a carbon disulfide solution and in the presence of AlCl_3_ [72]. The octadeuterated decaborane [μ_4_-1,2,3,4-B_10_H_6_D_8_] was prepared by the reaction of [1,2,3,4-B_10_H_10_D_4_] with D_2_O in acetonitrile [72], whereas the dodecadeuterated decaborane [μ_4_-1,2,3,4,5,7,8,10-B_10_H_2_D_12_] was obtained by heating [μ_4_-B_10_H_10_D_4_] with DCl in carbon disulfide in sealed ampoule in the presence of AlCl_3_ [72]. Deuterated aromatic solvents can also act as a source of deuterium. For example, heating decaborane(14) in benzene-*d*_6_ in the presence of AlCl_3_ under reflux leads to the the tetradeuterated decaborane [1,2,3,4-B_10_H_10_D_4_], while the reaction of decaborane(14) with AlCl_3_ in toluene-*d*_8_ at 5 °C results in the dideuterated decaborane [2,4-B_10_H_12_D_2_] [171]. The reaction of [1,2,3,4-B_10_H_10_D_4_] with AlCl_3_ in benzene leads to [1,3-B_10_H_12_D_2_] [172].

## 3. Halogen Derivatives

**Figure 3:**
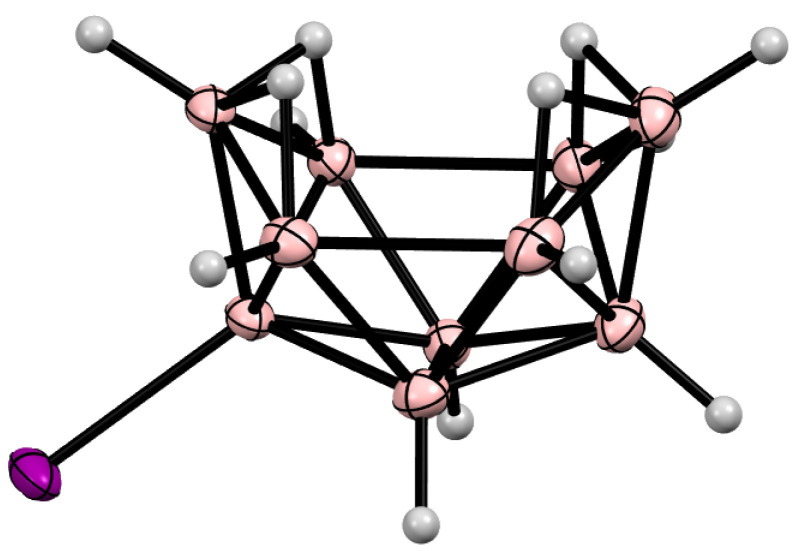
Solid state structure of the 2-iodo derivative of decaborane [2-I-B_10_H_13_].

Stock first reported the preparation of halogen derivatives of decaborane(14) by the direct reaction of decaborane with halogens in a sealed tube [33]. These reactions were re-investigated in the 1960s. It was found that the reaction of decaborane(14) with 1 equiv. iodine at 110–120 °C leads to the formation of a mixture of 1- and 2-iodo derivatives of decaborane in a ratio of ~1:2 [173,174]. The resulting mixture of isomers can be separated by fractional crystallization from low-boiling alkanes (pentane, hexane, heptane) [173] or chromatographically [175]. The assignment of the substitution position was made based on the ^11^B NMR spectra [174,176,177]; however, the 1-isomer was initially erroneously assigned as the 5-isomer [173,178]. The subsequent reaction of 2-iododecaborane with iodine at 110 °C results in a mixture of the 1,2- and 2,4-diiodo derivatives [1,2-I_2_-B_10_H_12_] and [2,4-I_2_-B_10_H_12_] in a nearly equal ratio, while the similar reaction of 1-iododecaborane produces mainly [1,2-I_2_-B_10_H_12_]. The reaction of decaborane(14) with an excess of iodine was found to give a mixture of [1,2-I_2_-B_10_H_12_] and [2,4-I_2_-B_10_H_12_] in a ratio of ~1:2 [173]. The reaction of decaborane(14) with iodine or iodine chloride in carbon disulfide in the presence of AlCl_3_ was found to give a mixture of the 1- and 2-iodo derivatives of decaborane in the same ratio of ~1:2 [179]. Later, the 1- and 2-iodo derivatives of decaborane were synthesized by the reaction of decaborane(14) with iodine chloride in refluxing dichloromethane in the presence of AlCl_3_ and reliably characterized by NMR spectroscopy [180]. The solid state structures of [1-I-B_10_H_13_] [181], [2-I-B_10_H_13_] (Figure 3) [180], and [2,4-I_2_-B_10_H_12_] [182] were determined by single-crystal X-ray diffraction. 

**Figure 4:**
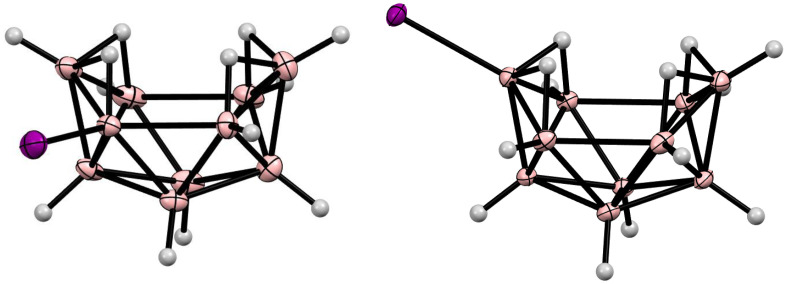
Solid state structures of [5-I-B_10_H_13_] (**left**) and [6-I-B_10_H_13_] (**right**).

The 5- and 6-iodo derivatives of decaborane were prepared in an indirect way. The treatment of [*arachno*-6,9-(Me_2_S)_2_-B_10_H_12_] with anhydrous HI in benzene under reflux conditions results in a mixture of the 5- and 6-iodo derivatives of decaborane [5-I-B_10_H_13_] and [6-I-B_10_H_13_] [174], which was separated by fraction crystallization from hexane [174] or chromatographically [175]. The reaction of [*arachno*-6,9-(Et_2_S)_2_-B_10_H_12_] with anhydrous HI in benzene at room temperature was found to give the 5-iodo derivative [5-I-B_10_H_13_] [183,184,185]. The reaction of (NH_4_)_2_[*closo*-B_10_H_10_] with anhydrous HCl in a mixture of AlI_3_ and 1-butyl-3-methylimidazolium iodide (bmimI) at 70 °C proceeds with the boron cage opening and results in the 6-iodo derivative of decaborane [6-I-B_10_H_13_] [186]. The 6-iodo derivative isomerizes to the 5-iodo derivative [5-I-B_10_H_13_] in the presence of a catalytic amount of triethylamine in toluene at 60 °C. It is assumed that the isomerization occurs through the transformation of [6-I-B_10_H_13_] into the [6-I-B_10_H_13_]^−^ anion, followed by its isomerization [187]. The 6-iodo derivative [6-I-B_10_H_13_] was also found to undergo photochemical isomerization to [5-I-B_10_H_13_] under UV-irradiation in a solution [187]. The solid state structures of [5-I-B_10_H_13_] [187] and [6-I-B_10_H_13_] [186] were determined by single-crystal X-ray diffraction (Figure 4). The 6-iodo derivative [6-I-B_10_H_13_] was also obtained by the reaction of (NH_4_)_2_[*closo*-B_10_H_10_] with AlI_3_, followed by the hydrolysis of the resulting intermediate [188]. 

The reactions of decaborane(14) with bromine in dichloromethane in the presence of AlCl_3_ [189] or AlBr_3_ [190], or in carbon disulfide in the presence of AlCl_3_ [174], lead to the formation of a mixture of 1- and 2-bromo derivatives of decaborane [1-Br-B_10_H_13_] and [2-Br-B_10_H_13_], which can be separated by fractional crystallization from hexane [173,189,190] or chromatographically [175]. 

**Figure 5:**
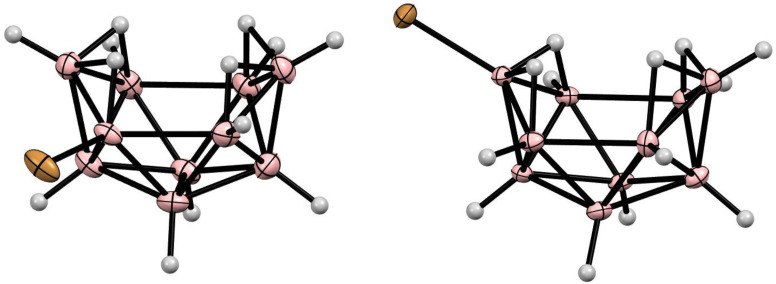
Solid state structures of [5-Br-B_10_H_13_] (**left**) and [6-Br-B_10_H_13_] (**right**).

The 5- and 6-bromo derivatives of decaborane were also prepared in an indirect way. The treatment of [*arachno*-6,9-(Me_2_S)_2_-B_10_H_12_] with anhydrous HBr in benzene under reflux conditions results in a mixture of the 5- and 6-bromo derivatives of decaborane [5-Br-B_10_H_13_] and [6-Br-B_10_H_13_] in a ratio of ~1:4, which was separated by fraction crystallization from hexane [174] or chromatographically [175,190]. The reactions of [*arachno*-6,9-(R_2_S)_2_-B_10_H_12_] (R = Me, Et) with anhydrous HBr in benzene at room temperature were found to give the 5-bromo derivative [5-Br-B_10_H_13_] [183,184,185]. The reaction of (NH_4_)_2_[*closo*-B_10_H_10_] with anhydrous HBr in a mixture of AlBr_3_ and 1-butyl-3-methylimidazolium bromide (bmimBr) at 70 °C proceeds with the boron cage opening and results in the 6-bromo derivative of decaborane [6-Br-B_10_H_13_] [186]. The 6-bromo derivative isomerizes to the 5-bromo derivative [5-Br-B_10_H_13_] in the presence of a catalytic amount of triethylamine in toluene at 60 °C [187]. The solid state structures of [5-Br-B_10_H_13_] [187] and [6-Br-B_10_H_13_] [186] were determined by single-crystal X-ray diffraction (Figure 5). The 6-bromo derivative [6-Br-B_10_H_13_] was also obtained by the reaction of (NH_4_)_2_[*closo*-B_10_H_10_] with AlBr_3_, followed by the hydrolysis of the resulting intermediate [188]. 

**Figure 6:**
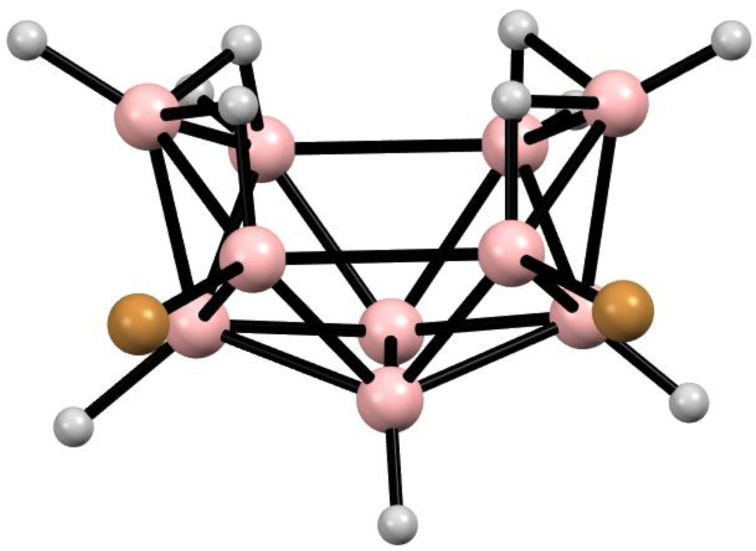
Solid state structure of the 5,10-dibromo derivative of decaborane [5,10-Br_2_-B_10_H_12_].

The reaction of dimethylstannaundecaborane [*nido*-Me_2_SnB_10_H_12_] with bromine in carbon disulfide leads to the oxidative removal of tin with the formation of the 5,10-dibromo derivative of decaborane [5,10-Br_2_-B_10_H_12_] (Figure 6) [191].

The reaction of decaborane(14) with chlorine in dichloromethane in the presence of AlCl_3_ results in a mixture of the 1- and 2-chloro derivatives of decaborane [1-Cl-B_10_H_13_] and [2-Cl-B_10_H_13_] [174,189]. Unexpectedly, a mixture of the 1- and 2-chloro derivatives of decaborane was obtained in the reaction of decaborane(14) with 1,1-difluoroethane in carbon disulfide in the presence of AlCl_3_ [192]. The isomers were separated by fractional crystallization from pentane or hexane [174,189,192] or chromatographically [175], and the substitution position was assigned using ^11^B NMR spectroscopy [174,193].

**Figure 7:**
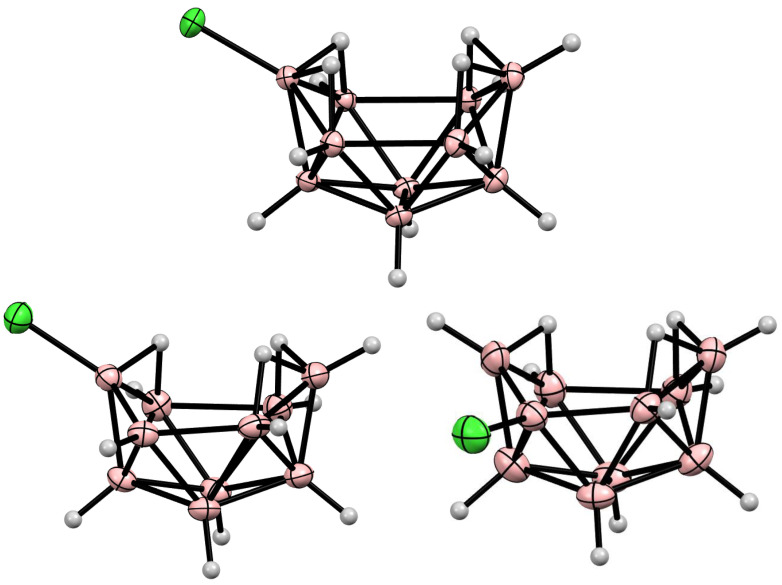
Solid state structures of [6-Cl-B_10_H_13_] (**top**) as well as the [6-Cl-B_10_H_12_]^−^ (**bottom left**) and [5-Cl-B_10_H_12_]^−^ (**bottom right**) anions in the crystal structures of the corresponding protonated Proton Sponge salts.

Similar to the corresponding iodo and bromo derivatives, the 5- and 6-chloro derivatives of decaborane were prepared in an indirect way. The treatment of [*arachno*-6,9-(Me_2_S)_2_-B_10_H_12_] with anhydrous HCl in benzene under reflux conditions results mainly in the 6-chloro derivative of decaborane [6-Cl-B_10_H_13_] with some amount of the 5-chloro isomer [174]. The reactions of [*arachno*-6,9-(Et_2_S)_2_-B_10_H_12_] with anhydrous HCl or HgCl_2_ in benzene at room temperature were also found to give the 6-chloro derivative [6-Cl-B_10_H_13_] [183,184,185]. The reaction of (NH_4_)_2_[*closo*-B_10_H_10_] with anhydrous HCl in a mixture of AlCl_3_ and 1-butyl-3-methylimidazolium chloride (bmimCl) at 70 °C proceeds with the boron cage opening and results in the 6-chloro derivative of decaborane [6-Cl-B_10_H_13_] [186]. The 6-chloro derivative of decaborane can also be prepared by the reactions of (NH_4_)_2_[*closo*-B_10_H_10_] with triflic acid in dichloromethane, respectively [186]. The 6-chloro derivative isomerizes to the 5-chloro derivative [5-Cl-B_10_H_13_] in the presence of a catalytic amount of triethylamine in toluene at 60 °C [187]. It was assumed that the isomerization occurs through the transformation of [6-Cl-B_10_H_13_] into the [6-Cl-B_10_H_13_]^−^ anion, followed by its isomerization. The solid state structures of [6-Cl-B_10_H_13_], (HPS)[6-Cl-B_10_H_12_], and (HPS)[5-Cl-B_10_H_12_] were determined by single-crystal X-ray diffraction (Figure 7) [186,187]. The B-H-B bridge deprotonation at the site adjacent to the halogenated boron atoms was revealed [187].

The 6-chloro derivative [6-Cl-B_10_H_13_] was also obtained by the reaction of (NH_4_)_2_[*closo*-B_10_H_10_] or (Et_4_N)_2_[*closo*-B_10_H_10_] with AlCl_3_, followed by the hydrolysis of the resulting intermediate [188,194,195].

**Figure 8:**
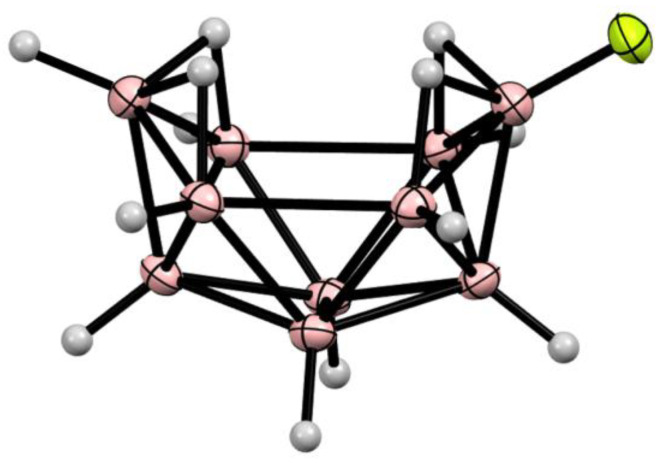
Solid state structure of the 6-fluoro derivative of decaborane [6-F-B_10_H_13_].

The 6-fluoro derivative of decaborane [6-F-B_10_H_13_] was first obtained by the reaction of [*arachno*-6,9-(Et_2_S)_2_-B_10_H_12_] with anhydrous HF in benzene at room temperature [183,184,185]. Later, the 6-fluoro derivative was prepared by the reaction of (NH_4_)_2_[*closo*-B_10_H_10_] with triflic acid in 1-fluoropentane [186]. The solid state structure of [6-F-B_10_H_13_] was determined by single-crystal X-ray diffraction (Figure 8) [186].

## 4. Derivatives with a B-O Bond

Due to the lack of electrophilic reagents for the introduction of oxygen substituents, the corresponding decaborane derivatives with substituents localized in the “bottom” of the decaborane basket have not yet been obtained. Alkoxy derivatives of decaborane [5-RO-B_10_H_13_] (R = Me, Et, Pr, Bu) were first obtained in low (13–20%) yields by trying to iodinate Na[B_10_H_13_] in the corresponding esters [196]. The phenoxy derivative [5-PhO-B_10_H_13_] was obtained in the same way, using anisole as a solvent [196]. The substitution position was determined using ^11^B NMR spectroscopy [197]. It was assumed that their formation proceeds through the formation of oxonium derivatives of *arachno*-decaborane [R_2_O-B_10_H_13_]^−^, followed by the elimination of one alkyl group [26]. The reaction of Na[B_10_H_13_] with SnCl_4_ in diethyl ether leads to a mixture of 6- and 5-alkoxy derivatives of decaborane [6-EtO-B_10_H_13_] and [5-EtO-B_10_H_13_] in a ratio varying from 85:15 to 70:30 depending on the reaction temperature. The isomers were separated using column chromatography on silica [198]. It should be noted that the direct reactions of decaborane(14) with alcohols and phenols ROH leads to its complete degradation to the corresponding trialkyl- or triarylborates (RO)_3_B [199]. The trimethylsiloxy derivative [6-Me_3_SiO-B_10_H_13_] was obtained in a low yield (5–20%) from the reactions of Na[B_10_H_13_] and Na_2_[B_10_H_12_] with Me_3_SiCl in diethyl ether [198]. The report on the preparation of a trimethylsilyl derivative [Me_3_Si-B_10_H_13_] under similar conditions [200] should apparently be considered erroneous.

**Figure 9:**
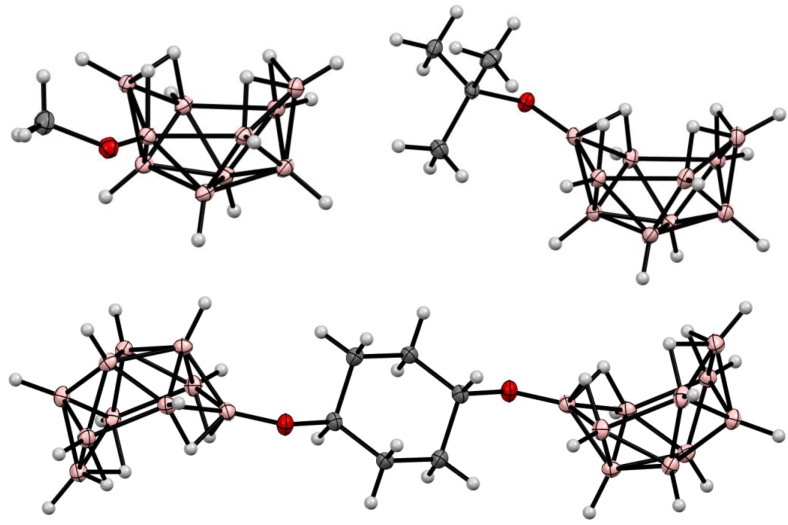
Solid state structures of [5-MeO-B_10_H_13_] (**top left**), [6-*t*-BuO-B_10_H_13_] (**top right**), and [μ-6,6′-(OC_6_H_10_O)-(B_10_H_13_)_2_] (**bottom**).

The reactions of 5-bromo derivative [5-Br-B_10_H_13_] with alcohols ROH in the presence of NaHCO_3_ in dichloromethane lead to 6-alkoxy derivatives [6-RO-B_10_H_13_] (R = Me, Et, *t*-Bu, *c*-Hx, CH_2_CH_2_SH, CH_2_CH_2_I, CH_2_CH_2_OCH_2_CH_2_Cl, (CH_2_)_3_C≡CH, CH(CH_2_CH=CH_2_)_2_), while the reactions of 6-bromo derivative [6-Br-B_10_H_13_] with alcohols ROH in the presence of NaHCO_3_ in dichloromethane lead to 5-alkoxy derivatives [5-RO-B_10_H_13_] (R = Me, *t*-Bu, cHx, CH_2_CH_2_SH, CH_2_CH_2_I, CH_2_CH_2_N(CO)_2_C_2_H_4_, CH_2_CH_2_OCH_2_CH_2_Cl, (CH_2_)_3_C≡CH, CH_2_C≡CCH_3_, CH(CH_2_CH=CH_2_)_2_). The reactions of [5-Br-B_10_H_13_] and [6-Br-B_10_H_13_] with 1,4-cyclohexyldiol lead to the compounds [μ-6,6′-(OC_6_H_10_O)-(B_10_H_13_)_2_] and [μ-5,5′-(OC_6_H_10_O)-(B_10_H_13_)_2_], respectively. The reactions of alcohols with [6-Br-B_10_H_13_] proceed quickly at room temperature, while those with [5-Br-B_10_H_13_] require heating (70 °C) to achieve completion. The reaction of [6-Br-B_10_H_13_] with ethanol was largely complete after 12 h at room temperature, but the reactions with 2-iodoethanol (~20 h), 2-bromoethanol (~40 h), 2-chloroethanol (~100 h), and 2-fluoroethanol (~125 h) all took increasingly longer times. The reactions with chloro- and iodo-derivatives of decaborane were found to proceed in a similar way; however, the reaction rate decreases in the halogen series I~Br > Cl [198]. The solid state structures of [5-MeO-B_10_H_13_] (Figure 9), [6-*t*-BuO-B_10_H_13_] (Figure 9), [5-ClCH_2_CH_2_OCH_2_CH_2_O-B_10_H_13_], [6-ClCH_2_CH_2_OCH_2_CH_2_O-B_10_H_13_], [5-MeC≡CCH_2_O-B_10_H_13_], and [μ-6,6′-(OC_6_H_10_O)-(B_10_H_13_)_2_] (Figure 9) were determined by single-crystal X-ray diffraction [201].

**Figure 10:**
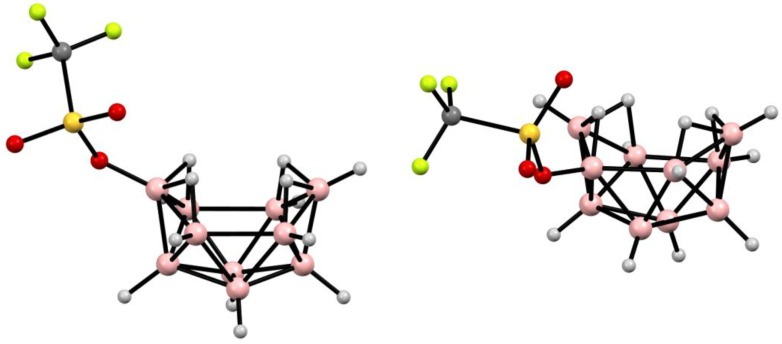
Solid state structures of [6-TfO-B_10_H_13_] (**left**) and [5-TfO-B_10_H_13_] (**right**).

The 6-triflate derivative of decaborane [6-TfO-B_10_H_13_] was prepared by the reaction of Cs_2_[*closo*-B_10_H_10_] with neat triflic acid at an ambient temperature [202,203]. In contrast, the reaction of (NH_4_)_2_[*closo*-B_10_H_10_] with triflic acid in 1-butyl-3-methylimidazolium triflate at 60 °C results in the 5-triflate derivative of decaborane [5-TfO-B_10_H_13_] [204]. It was found that the reaction proceeds through the formation of the 6-triflate derivative, which, upon heating, isomerizes into the 5-triflate derivative. In the presence of a catalytic amount of triethylamine, the isomerization of [6-TfO-B_10_H_13_] to [5-TfO-B_10_H_13_] proceeds even at room temperature [204]. The reactions of [5-TfO-B_10_H_13_] with methanol and 4-methoxyphenol in 1,2-dichloroethane at 70 °C result in the corresponding ethers [6-RO-B_10_H_13_] (R = Me, C_6_H_4_-4-OMe) [204]. The solid state structures of [6-TfO-B_10_H_13_] [202] and [5-TfO-B_10_H_13_] [204] were determined by single-crystal X-ray diffraction (Figure 10).

The reactions of Na_2_[*closo*-B_10_H_10_] with alcohols ROH (R = Me, Et, *i-*Pr, Bu, Ph) in hexane in the presence of trimethylsilyl triflate lead to the corresponding 6-alkoxy derivatives of decaborane [6-RO-B_10_H_13_] [205]. The reaction with water under the same conditions results in the 6-trimethylsiloxy derivative [6-Me_3_SiO-B_10_H_13_] [205]. 

In a similar way, the reaction of (NH_4_)_2_[*closo*-B_10_H_10_] with sulfuric acid produces the 6-hydroxy derivative of decaborane [6-HO-B_10_H_13_] [206]. The 6-hydroxy derivative was also obtained as a by-product of the reaction of [*arachno*-6,9-(Me_2_S)_2_-B_10_H_12_] with sulfuric acid in benzene [207].

**Figure 11:**
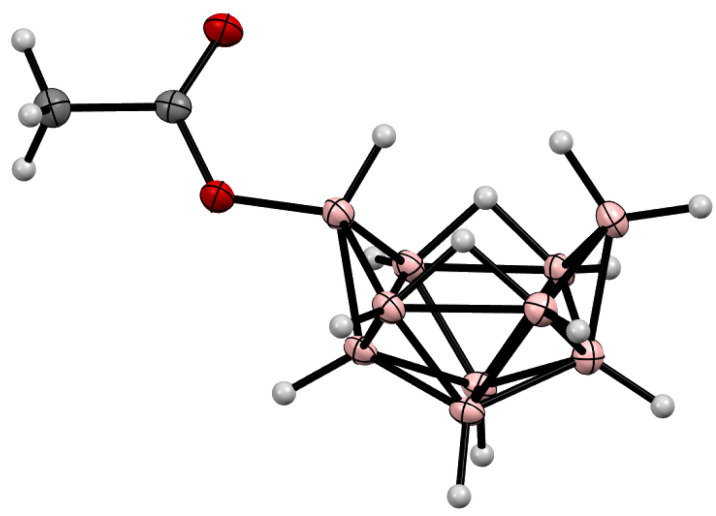
Structure of the [6-*arachno*-AcO-B_10_H_13_]^2−^ anion in the crystal structure of (C_2_mim)_2_[6-AcO-B_10_H_13_].

The 6-acetoxy derivative of *nido*-decaborane [6-AcO-B_10_H_13_] was obtained by the reaction of [*arachno*-6,9-(Me_2_S)_2_-B_10_H_12_] with mercury acetate [208]. The 6-acetoxy derivative of the *arachno*-decaborate anion [*arachno*-6-AcO-B_10_H_13_]^2−^ was obtained by the reaction of decaborane with 1-ethyl-3-methylimidazolium acetate (C_2_mim)(OAc). The solid state structure of (C_2_mim)_2_[6-AcO-B_10_H_13_] was determined by single-crystal X-ray diffraction (Figure 11) [209].

The bis(decaboranyl) ether [μ-6,6′-O-(B_10_H_13_)_2_] was prepared by the reaction of [*arachno*-6,9-(R_2_S)_2_-B_10_H_12_] (R = Me, Et) with sulfuric acid in benzene [210,211]. Its structure was determined by ^11^B NMR spectroscopy [183,211] and supported by single-crystal X-ray diffraction [212]. The bis(decaboranyl) ether [μ-6,6′-O-(B_10_H_13_)_2_] was also obtained by the dehydration of (H_3_O)_2_[*closo*-B_10_H_10_] [213].

The preparation of decaborane derivatives with amides [*arachno*-6,9-(MeRN(R′)CO)_2_-B_10_H_12_] (R = H, R′ = H, Me; R = Me, R′ = H, Me), triphenylphosphine oxide [*arachno*-6,9-(Ph_3_PO)_2_-B_10_H_12_], and dimethylsulfoxide [*arachno*-6,9-(Me_2_SO)_2_-B_10_H_12_] has also been reported [214,215,216,217]. 

## 5. Derivatives with a B-S Bond

**Figure 12:**
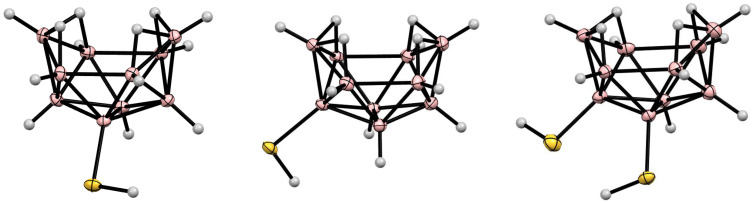
Solid state structures of the mercapto derivatives of decaborane [1-HS-B_10_H_13_] (**left**), [2-HS-B_10_H_13_] (**middle**), and [1,2-(HS)_2_-B_10_H_12_] (**right**).

The reaction of decaborane(14) with sulfur in the presence of AlCl_3_ at 120 °C results in a mixture of the mercapto derivatives [1-HS-B_10_H_13_], [2-HS-B_10_H_13_], and [1,2-(HS)_2_-B_10_H_12_] [218,219]. The solid state structures of these mercapto derivatives were determined by single-crystal X-ray diffraction (Figure 12) [219].

The reactions of Na_2_[*closo*-B_10_H_10_] with thiols RSH (R = *i-*Pr, *i*-Bu, *c*-Hx, C_6_H_4_-*p*-Me, C_6_H_4_-*p*-F) in hexane in the presence of trimethylsilyl triflate led to the corresponding 6-alkyl- and 6-arylsulfides [6-RS-B_10_H_13_] [205]. It should be noted that the direct reactions of decaborane(14) with alkyl thiols RS lead to its complete degradation to the corresponding trialkylthioborates (RS)_3_B [220].

Due to its use in the synthesis of carboranes, the 6,9-bis(dimethylsulfonium) derivative of the *arachno*-decaborate anion [*arachno*-6,9-(Me_2_S)_2_-B_10_H_12_], which is formed by refluxing decaborane with dimethyl sulfide in ether or benzene, is the most known decaborane derivative with a B-S bond [214,221,222]. The solid state structure of [*arachno*-6,9-(Me_2_S)_2_-B_10_H_12_] was determined by single-crystal X-ray diffraction [223]. The 6,9-bis(dimethylsulfonium) derivative was studied by X-ray and X-ray photoelectron spectroscopy [224,225,226], and its diamagnetic susceptibility was determined [227]. The Me_2_S substituents in [*arachno*-6,9-(Me_2_S)_2_-B_10_H_12_] can be easily replaced by stronger Lewis bases [214,228]. A series of other bis(dialkylsulfonium) derivatives ([*arachno*-6,9-(RR′S)_2_-B_10_H_12_] (R = R′ = Et, Pr; RR′ = (CH_2_)_4_, (CH_2_CH_2_)_2_S, (CH_2_CH_2_)_2_O)) have been prepared in a similar manner [215,220,228,229].

The bis(diethylsulfonium) derivative [*arachno*-6,9-(Et_2_S)_2_-B_10_H_12_] can also be obtained by the reaction of (NH_4_)_2_[*closo*-B_10_H_10_] with anhydrous hydrogen chloride in diethylsulfide [230]. This approach has been extended to other salts of the *closo*-decaborate anion and other strong acids, including (H_3_O)_2_[*closo*-B_10_H_10_] [231,232].

The reactions of 2-halogen derivatives of decaborane [2-X-B_10_H_13_] (X = Cl, Br, I) with dimethylsulfide Me_2_S give the corresponding 2-halogen-6,9-bis(dimethylsulfonium) derivatives [*arachno*-2-X-6,9-(Me_2_S)_2_-B_10_H_11_] [233,234]. In a similar way, the reactions of 5-halogen derivatives of decaborane [5-X-B_10_H_13_] (X = F, Br, I) with dialkylsulfides R_2_S (R = Me, Et) result in the corresponding 5-halogen-6,9-bis(dialkylsulfonium) derivatives [*arachno*-5-X-6,9-(R_2_S)_2_-B_10_H_11_], while the reactions of the 6-chloro derivative proceed with the halogen displacement, giving [*arachno*-6,9-(R_2_S)_2_-B_10_H_12_] [185]. The solid state structure of [5-Br-6,9-(R_2_S)_2_-B_10_H_11_] was determined by single-crystal X-ray diffraction [235]. The reactions of 2,4-dichloro- and 1,2,4-trichloro derivatives of decaborane [*nido*-2,4-Cl_2_-B_10_H_12_] and [*nido*-1,2,4-Cl_3_-B_10_H_11_] with dimethylsulfide were found to proceed with the boron cage rearrangement, resulting in [*arachno*-1,7-Cl_2_-6,9-(Me_2_S)_2_-B_10_H_10_] and [*arachno*-1,3,7-Cl_3_-6,9-(Me_2_S)_2_-B_10_H_9_], respectively [236]. The solid state structures of [1,7-Cl_2_-6,9-(Me_2_S)_2_-B_10_H_10_] and [1,3,7-Cl_3_-6,9-(Me_2_S)_2_-B_10_H_9_] were determined by single-crystal X-ray diffraction [236].

The reaction of the 5-triflato derivative of decaborane [5-TfO-B_10_H_13_] with dimethylsulfide in toluene results in the 5-triflato-6,9-bis(dialkylsulfonium) derivative [*arachno*-5-TfO-6,9-B_10_H_11_(SMe_2_)_2_], while the similar reaction of the 5-triflato derivative [6-TfO-B_10_H_13_] proceeds with the substitution of the triflate group, giving [*arachno*-6,9-B_10_H_12_(SMe_2_)_2_] [204]. The solid state structure of [5-TfO-6,9-(Me_2_S)_2_-B_10_H_11_] was determined by single-crystal X-ray diffraction [204]. 

**Figure 13:**
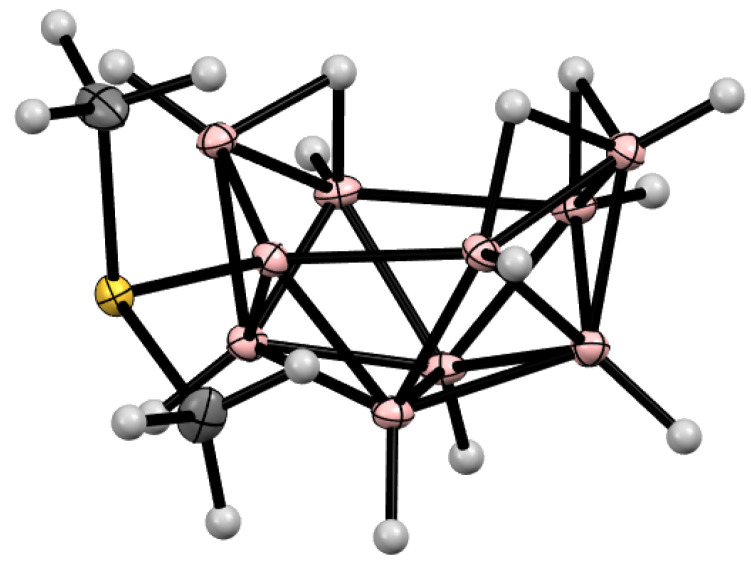
Solid state structure of [*nido*-5-Me_2_S-B_10_H_12_].

The 5-dimethylsulfonium derivative of decaborane [*nido*-5-Me_2_S-B_10_H_12_] was obtained by heating [*arachno*-6,9-(Me_2_S)_2_-B_10_H_12_] in toluene or mesitylene, and its solid state structure was determined by single-crystal X-ray diffraction (Figure 13) [215,222,237,238]. It was found that the B-H-B bridge deprotonation occurs at the site adjacent to the substituted boron atom, and thus, the structure of [5-Me_2_S-B_10_H_12_] is similar to the structure of the [6-Cl-B_10_H_12_]^−^ anion [187].

The preparation of the bis(dimethylthioformamide) [*arachno*-6,9-(Me_2_N(H)CS)_2_-B_10_H_12_] [215] and the bis(di(alkyl/aryl)thiourea) [*arachno*-6,9-((RHN)_2_CS)_2_-B_10_H_12_] (R = Et, Ph) [217,239] derivatives has also been reported. 

## 6. Derivatives with a B-N Bond

**Figure 14:**
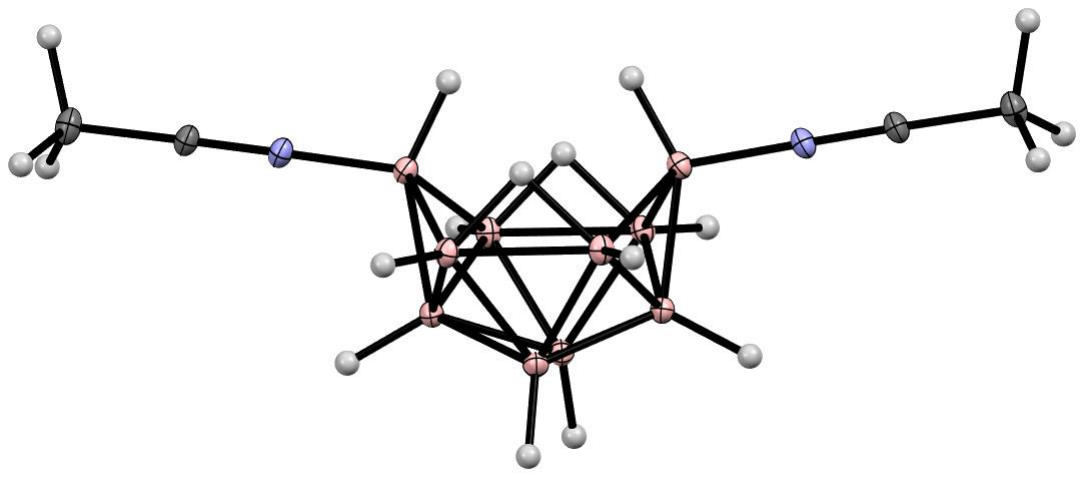
Solid state structure of [*arachno*-6,9-(MeC≡N)_2_-B_10_H_12_].

Heating decaborane(14) in acetonitrile under reflux proceeds with hydrogen elimination, resulting in the 6,9-bis(acetonitrilium) derivative of the *arachno*-decaborate anion [*arachno*-6,9-(MeC≡N)_2_-B_10_H_12_] [240], which was the first structurally characterized decaborane derivative [217,241,242,243] (Figure 14). The 6,9-bis(acetonitrilium) derivative was studied by X-ray photoelectron spectroscopy [226], and its diamagnetic susceptibility was determined [227]. The 6,9-bis(propionitrilium) and 6,9-bis(benzonitrilium) derivatives [*arachno*-6,9-(RC≡N)_2_-B_10_H_12_] (R = Et, Ph) were prepared in a similar way from decaborane(14) and propionitrile [244] or benzonitrile [217], respectively. The reaction of the 6,9-bis(acetonitrilium) derivative with diethylcyanamide in diethyl ether results in [*arachno*-6,9-(Et_2_NC≡N)_2_-B_10_H_12_] [214,245]. The reaction of the 2-bromo derivative of decaborane [2-Br-B_10_H_13_] gives [*arachno*-6,9-(MeC≡N)_2_-2-Br-B_10_H_11_] [233].

**Figure 15:**
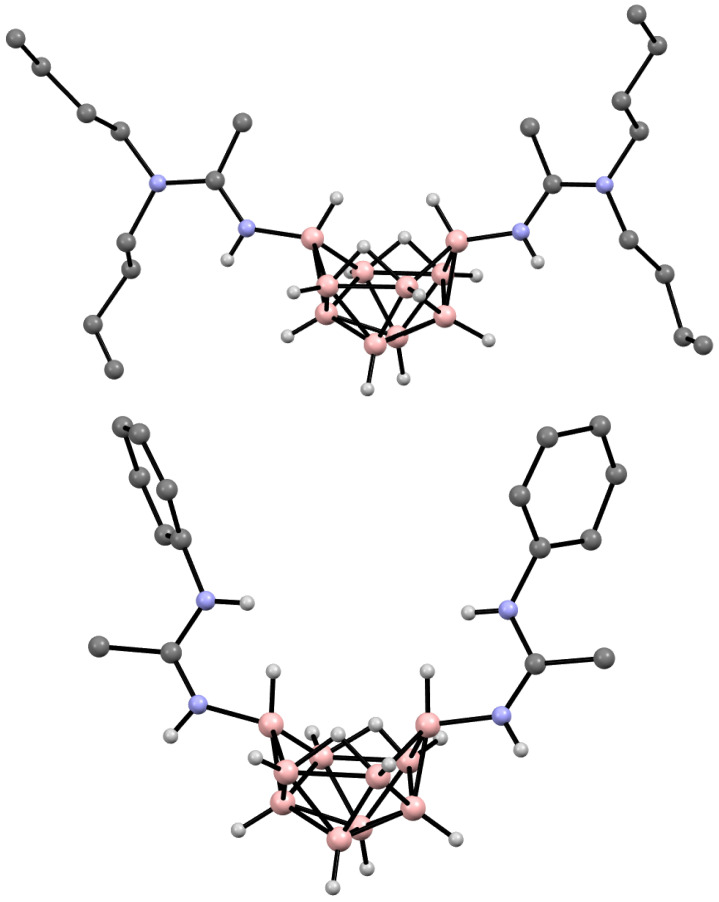
Solid state structures of the decaborane-based amidines [6,9-(Bu_2_N(Me)C=HN)_2_-B_10_H_12_] (**top**) and [6,9-(PhHN(Me)C=HN)_2_-B_10_H_12_] (**bottom**).

The 6,9-bis(acetonitrilium) derivative [*arachno*-6,9-(MeC≡N)_2_-B_10_H_12_] reacts with *N*-nucleophiles (primary and secondary amines and hydrazine), giving the corresponding amidines [*arachno*-6,9-(RR′N(Me)C=HN)_2_-B_10_H_12_] (R = H, R′ = Et, Pr, Bu, Ph, NH_2_, NHMe; R = R′ = Et, Pr, Bu] [246,247,248]. The solid state structures of the amidines [6,9-(Bu_2_N(Me)C=HN)_2_-B_10_H_12_] and [6,9-(PhHN(Me)C=HN)_2_-B_10_H_12_]·Et_2_O were determined by single-crystal X-ray diffraction (Figure 15) [248]. It should be noted that the reactions with primary amines produce mainly the *ZE* isomers, whereas the reactions with secondary amines result only in the *EE* isomers.

The reaction of the 6,9-bis(acetonitrilium) derivative with methanol results in the formation of the corresponding imidate [*arachno*-6,9-(MeO(Me)C=HN)_2_-B_10_H_12_] [248]. Nowadays, the addition of nucleophiles to the activated triple -C≡N- bond of nitrilium derivatives of various polyhedral boron hydrides has become a widely used method for their modification [249,250,251,252,253,254,255,256,257,258].

**Figure 16:**
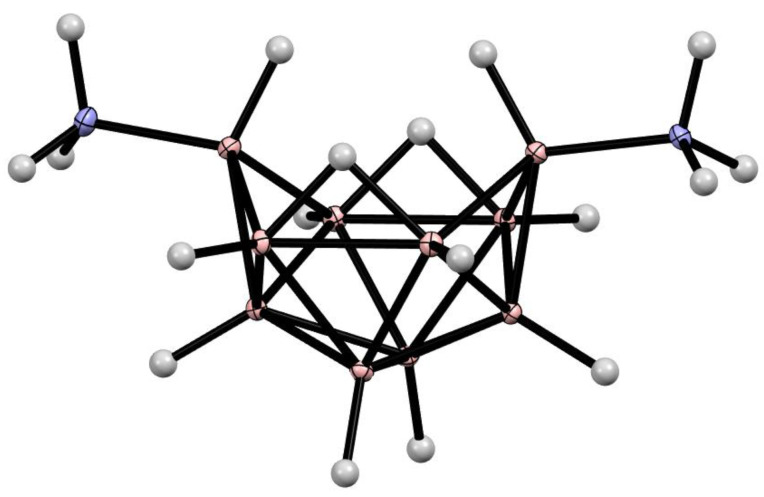
Solid state structure of [*arachno*-6,9-(H_3_N)_2_-B_10_H_12_].

The reactions of the 6,9-bis(acetonitrilium) derivative with tertiary amines in refluxing benzene or toluene lead to the corresponding 6,9-bis(trialkylammonium) derivatives [*arachno*-6,9-(R_3_N)_2_-B_10_H_12_] (R = Me, Et) [214,245]. The 6,9-bis(ammonium) derivative [*arachno*-6,9-(H_3_N)_2_-B_10_H_12_] was prepared by the reaction of decaborane(14) with ammonia in benzene or toluene [259,260]. The solid state structures of [6,9-(H_3_N)_2_-B_10_H_12_] (Figure 16) [243,261], [6,9-(Me_3_N)_2_-B_10_H_12_] [262], and [6,9-(Et_3_N)_2_-B_10_H_12_] [263] were determined by single-crystal X-ray diffraction. [6,9-(H_3_N)_2_-B_10_H_12_] and [6,9-(Et_3_N)_2_-B_10_H_12_] were studied by X-ray photoelectron and X-ray fluorescence spectroscopy [225,226,264]. The diamagnetic susceptibilities of [6,9-(Me_3_N)_2_-B_10_H_12_] and [6,9-(Et_3_N)_2_-B_10_H_12_] were determined [227]. The thermal decomposition of the 6,9-bis(ammonium) derivative [*arachno*-6,9-(H_3_N)_2_-B_10_H_12_] was studied [260,265,266].

The reaction of decaborane(14) with diethylamine in cyclohexane results in the diethylammonium derivative of the *arachno*-decaborate anion (Et_2_NH_2_)[*arachno*-6-Et_2_HN-B_10_H_13_] [267]. Similar alkylammonium derivatives (Me_4_N)[*arachno*-6-RR′R″N-B_10_H_13_] (R = Et, R′ = R″ = H; R = R′ = Et, R″ = H; R = R′ = R″ = Et; RR′ = (CH_2_)_5_, R″ = H) were prepared by the reactions of Na[B_10_H_13_] with the corresponding amines, followed by precipitation with (Me_4_N)Cl [267]. Heating (Et_2_NH_2_)[*arachno*-6-Et_2_HN-B_10_H_13_] in THF under reflux gives the 6,9-bis(diethylammonium) derivative [*arachno*-6,9-(Et_2_HN)_2_-B_10_H_12_], whereas the similar reaction in acetonitrile leads to [*arachno*-6-Et_2_HN-9-Et_2_N(Me)C=HN-B_10_H_12_] [267]. The reactions of Na[*arachno*-6-Et_2_HN-B_10_H_13_] with acetonitrile and dimethylsulfide in the presence of dry HCl result in [*arachno*-6-Et_2_NH-9-MeC≡N-B_10_H_12_] and [*arachno*-6-Et_2_NH-9-Me_2_S-B_10_H_12_], respectively [267]. In a similar way, the reaction of (Me_4_N)[*arachno*-6-Et_3_N-B_10_H_13_] with acetonitrile leads to [*arachno*-6-Et_3_N-9-MeC≡N-B_10_H_12_] [221]. The reactions of Na[*arachno*-6-Et_2_HN-B_10_H_13_] with amines in THF produce the corresponding 6,9-bis(alkylammonium) derivatives [*arachno*-6-Et_2_NH-9-RR′R′’N-B_10_H_12_] (R = Et, R′ = R″ = H; R = R′ = Et, R″ = H; R = R′ = R″ = Me) [267]. 

**Scheme 4:**
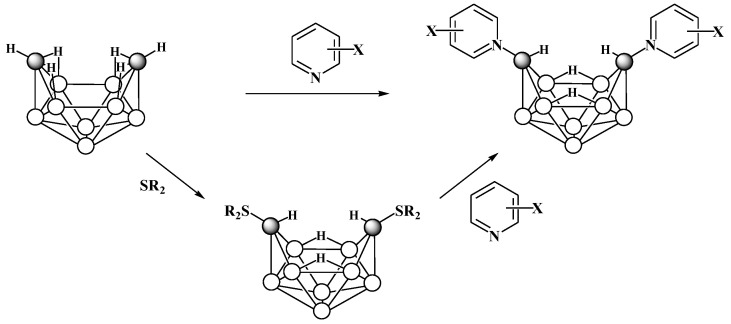
Synthesis of 6,9-bis(pyridinium) derivatives [6,9-L_2_-B_10_H_12_].

The reactions of decaborane(14) with pyridines, quinolines, and isoquinoline lead to the corresponding [*arachno*-6,9-L_2_-B_10_H_12_] (L = pyridine, 2-methylpyridine, 3-methylpyridine, 4-methylpyridine, 2-ethynylpyridine, 2-cyanopyridine, quinoline, 2-methylquinoline, 8-methylquinoline, isoquinoline) derivatives (Scheme 4) [214,236,268,269]. A more convenient way to prepare 6,9-bis(pyridinium) derivatives is the nucleophilic substitution of the dialkylsulfide groups in [*arachno*-6,9-(R_2_S)_2_-B_10_H_12_] (R = Me, Et). In this way, the [*arachno*-6,9-L_2_-B_10_H_12_] (L = pyridine, 2-methylpyridine, 3-methylpyridine, 4-methylpyridine, 2,3-dimethylpyridine, 2,4-dimethylpyridine, 2,5-dimethylpyridine, 2,6-dimethylpyridine, 3,4-dimethylpyridine, 2-methyl-5-ethylpyridine, 2-phenylpyridine, 4-benzylpyridine, 4-styrylpyridine, 2-methoxypyridine, 4-methoxypyridine, 3-chloropyridine, 4-chloropyridine, 2-bromopyridine, 4-bromopyridine, 3,5-dibromopyridine, 3-cyanopyridine, 4-cyanopyridine, 4-acetylpyridine, quinoline) derivatives were synthesized (Scheme 4) [229,270,271,272].

**Figure 17:**
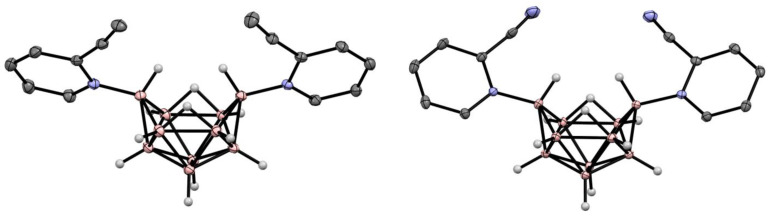
Solid state structures of [*arachno*-6,9-(HC≡C-*o*-C_5_H_4_N)_2_-B_10_H_12_] (**left**) and [*arachno*-6,9-(N≡C-*o*-C_5_H_4_N)_2_-B_10_H_12_] (**right**). Hydrogen atoms of organic substituents are omitted for clarity.

All compounds of this series are brightly colored from yellow to red, which is the reason for the interest in their study by UV and luminescent spectroscopy [229,271,272,273]. The 6,9-bis(pyridinium) derivative [6,9-Py_2_-B_10_H_12_] was studied by X-ray photoelectron and X-ray fluorescence spectroscopy [225,226] and, its diamagnetic susceptibility was determined [227]. The thermal decomposition of the 6,9-bis(pyridinium) and 6,9-bis(quinolinium) derivatives was studied [274]. The solid state structures of [6,9-Py_2_-B_10_H_12_] [275], [6,9-(HC≡C-*o*-C_5_H_4_N)_2_-B_10_H_12_] [269], and [6,9-(N≡C-*o*-C_5_H_4_N)_2_-B_10_H_12_]·CH_2_Cl_2_ [269] were determined by single-crystal X-ray diffraction (Figure 17).

**Scheme 5:**
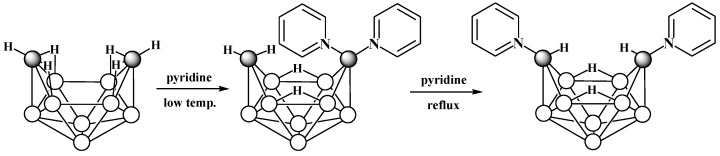
Synthesis of [*arachno*-6,6-Py_2_-B_10_H_12_] and its transformation to [*arachno*-6,9-Py_2_-B_10_H_12_].

It should be noted that the reaction of decaborane(14) with pyridine at low temperatures was found to form the 6,6-bis(pyridinium) derivative [*arachno*-6,6-Py_2_-B_10_H_12_], which, upon refluxing in dry degassed pyridine, converts into the more stable 6,9-isomer (Scheme 5) [268]. 

**Figure 18:**
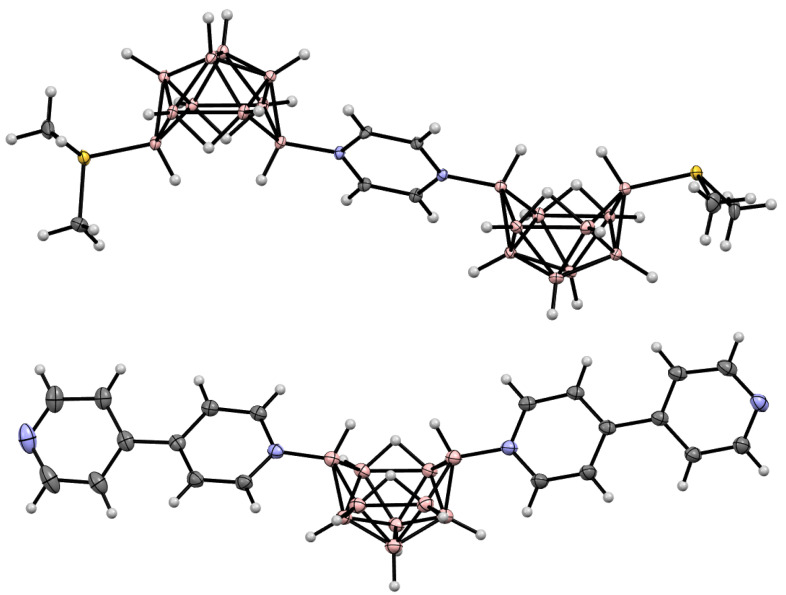
Solid state structures of [μ-6,6′-pyrazine-(9-Me_2_S-B_10_H_12_)_2_] (**top**) and [6,9-(NC_5_H_4_C_5_H_4_N)_2_-B_10_H_12_] (**bottom**).

The reaction of [6,9-(Me_2_S)_2_-B_10_H_12_] with pyrazine in dichloromethane gives the pyrazine-bridged derivative [μ-6,6′-pyrazine-(9-Me_2_S-B_10_H_12_)_2_], whereas the reaction with 4,4′-bipyridine leads to the product of the substitution of the Me_2_S groups with azaheterocycle [6,9-(NC_5_H_4_C_5_H_4_N)_2_-B_10_H_12_] (Figure 18) [276]. 

**Scheme 6:**
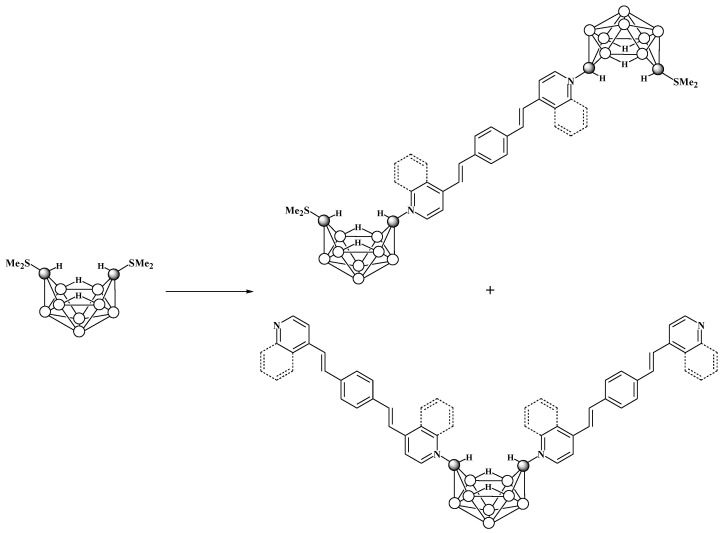
Reactions of [*arachno*-6,9-(Me_2_S)_2_-B_10_H_12_] with 1,4-bis[β-(4-pyridyl)vinyl]benzene and 1,4-bis[β-(4-quinolyl)vinyl]benzene.

The similar reactions of [6,9-(Me_2_S)_2_-B_10_H_12_] with 1,4-bis[β-(4-pyridyl)vinyl]benzene and 1,4-bis[β-(4-quinolyl)vinyl]benzene were found to produce mixtures of the corresponding bridged and terminal substituted derivatives (Scheme 6) [277].

The reactions of Na[*arachno*-6-Et_2_HN-B_10_H_13_] and (Me_4_N)[*arachno*-6-Et_3_N-B_10_H_13_] with pyridine in THF produce the corresponding 6-alkylammonium-9-pyridinium derivatives [*arachno*-6-Et_2_RN-9-Py-B_10_H_12_] (R = H, Et) [267,278].

**Figure 19:**
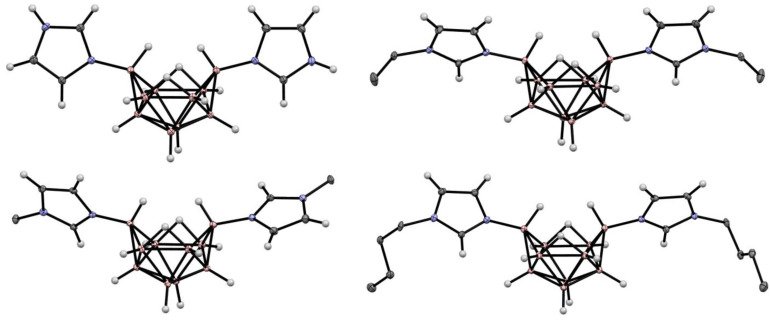
Solid state structures of [6,9-(HIm)_2_-Me_2_S-B_10_H_12_] (**top left**), [6,9-(MeIm)_2_-Me_2_S-B_10_H_12_] (**top right**), [6,9-(EtIm)_2_-Me_2_S-B_10_H_12_] (**bottom left**), and [6,9-(BuIm)_2_-Me_2_S-B_10_H_12_] (**top right**). Hydrogen atoms of alkyl groups are omitted for clarity.

The reactions of decaborane(14) with imidazoles in refluxing benzene result in the corresponding 6,9-bis(imidazolium) derivatives [*arachno*-6,9-(RIm)_2_-Me_2_S-B_10_H_12_] (R = H, Me, Et, Bu) (Figure 19). The hypergolic properties of the 6,9-bis(imidazolium) derivatives prepared were studied [279].

The reactions of decaborane(14) with 2-isopropyl- and 2-methyl-5-(2-chloroethyl) tetrazoles in benzene result in the corresponding 6,9-bis(tetrazolium) derivatives [*arachno*-6,9-L_2_-Me_2_S-B_10_H_12_] [280].

**Figure 20:**
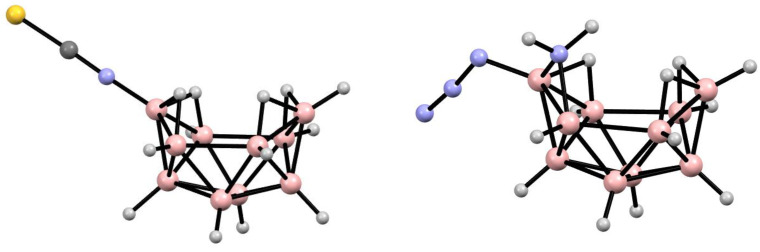
Solid state structures of [6-SCN-B_10_H_13_] (**left**) and [6-N_3_-μ-5,6-NH_2_-B_10_H_11_] (**right**).

The 6-isothiocyanato derivative [6-SCN-B_10_H_13_] was prepared by the reaction of [6,9-(R_2_S)_2_-B_10_H_12_] (R = Me, Et) with mercury isothiocyanate [208]. Alternatively, the 6-isothiocyanato derivative can be prepared by the reaction of decaborane(14) with NaSCN in 1,2-dimethoxyethane in the presence of dry HCl [281]. The solid state structure of [6-SCN-B_10_H_13_] was determined by single-crystal X-ray diffraction (Figure 20) [281].

The reaction of [6,9-(Me_2_S)_2_-B_10_H_12_] with excess HN_3_ in toluene results in the 6-azido-μ-5,6-amino derivative [6-N_3_-μ-5,6-NH_2_-B_10_H_11_], the structure of which was determined by single-crystal X-ray diffraction (Figure 20) [282].

## 7. Derivatives with a B-P Bond

The reaction of decaborane(14) with triphenylphosphine in diethyl ether under reflux results in the 6,9-bis(triphenylphosphonium) derivative of the *arachno*-decaborate anion [*arachno*-6,9-(Ph_3_P)_2_-B_10_H_12_]; the same product can be prepared by the reaction of [6,9-(MeC≡N)_2_-B_10_H_12_] with triphenylphosphine in hot acetonitrile [214,245,283]. The solid state structure of [*arachno*-6,9-(Ph_3_P)_2_-B_10_H_12_]·2DMF·H_2_O was determined by single-crystal X-ray diffraction [284]. The 6,9-bis(triphenylphosphonium) derivative was also studied by X-ray emission and X-ray photoelectron spectroscopy [279,280], and its diamagnetic susceptibility was determined [227]. The reaction of [6,9-(Me_2_S)_2_-2-Br-B_10_H_11_] with triphenylphosphine in benzene results in the corresponding 6,9-bis(triphenylphosphonium) derivative [6,9-(Ph_3_P)_2_-2-Br-B_10_H_11_] [233].

**Figure 21:**
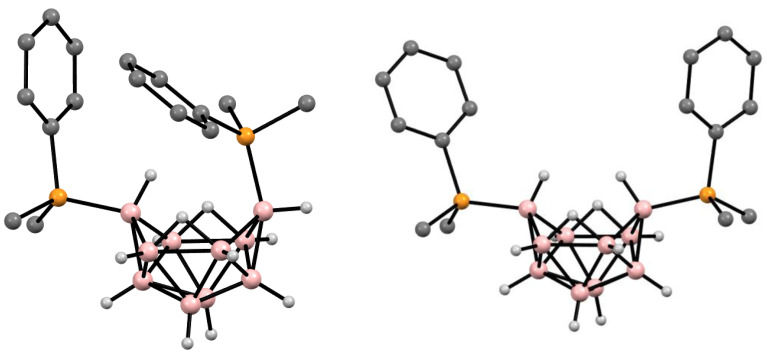
Solid state structures of *exo,endo-*[6,9-(PhMe_2_P)_2_-*arachno*-B_10_H_12_] (**left**) and *exo,exo-*[6,9-(PhMe_2_P)_2_-*arachno*-B_10_H_12_] (**right**). Hydrogen atoms of organic substituents are omitted for clarity.

The reaction of decaborane(14) with PhMe_2_P at low temperatures (~200 K) gives a mixture of *exo,exo-* and *exo,endo-*isomers of [6,9-(PhMe_2_P)_2_-*arachno*-B_10_H_12_], which were separated chromatographically [285,286]. The structure of both isomers was confirmed by single-crystal X-ray diffraction (Figure 21) [286].

**Figure 22:**
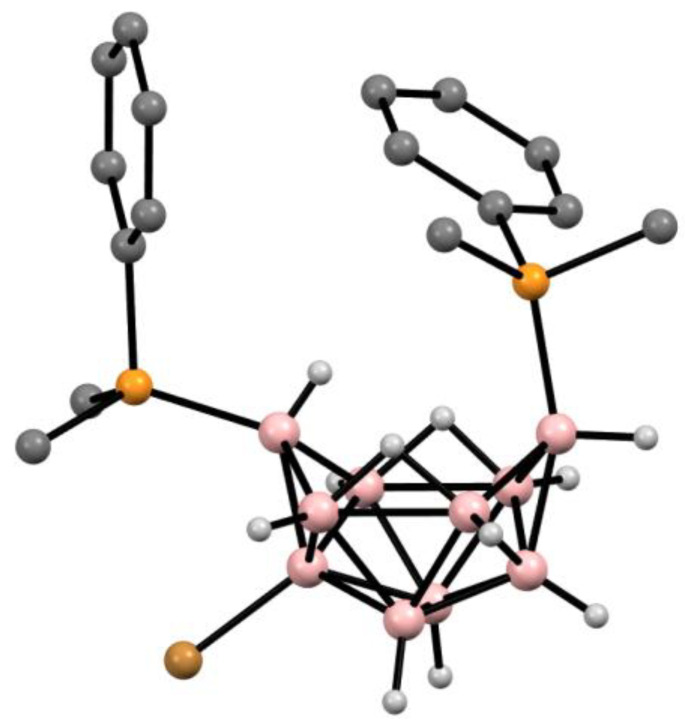
Solid state structure of *exo,endo-*[6,9-(PhMe_2_P)_2_-2-Br-*arachno*-B_10_H_12_]. Hydrogen atoms of organic substituents are omitted for clarity.

Under similar conditions, the reaction of the 2,4-dichloro derivatives of decaborane with PhMe_2_P solely produces the *exo,endo-*isomer of [6,9-(PhMe_2_P)_2_-2,4-Cl_2_-*arachno*-B_10_H_10_], while the reaction of 2-bromo leads to mixtures of *exo,exo-* and *exo,endo-*isomers and [6,9-(PhMe_2_P)_2_-2-Br-*arachno*-B_10_H_11_]. It is interesting to note that in the reaction of the 2-bromo derivative, it is precisely the 6,9-*exo,endo-*isomer that is formed, without any traces of the 9,6-*exo,endo-*isomer. The solid state structure of the *exo,endo-*isomer [6,9-(PhMe_2_P)_2_-2-Br-*arachno*-B_10_H_12_] was determined by single-crystal X-ray diffraction (Figure 22) [286]. 

The reaction of decaborane(14) with triethylphosphine in benzene results in the 6,9-bis(triethylphosphonium) derivative [*arachno*-6,9-(Et_3_P)_2_-B_10_H_12_] [287], whereas the 6,9-bis(diphenylphosphonium) and 6,9-bis(phenylphosphonium) derivatives [*arachno*-6,9-(Ph_2_HP)_2_-B_10_H_12_] and [*arachno*-6,9-(PhH_2_P)_2_-B_10_H_12_] were prepared by the reactions of the corresponding phosphines with [*arachno*-6,9-(Et_2_S)_2_-B_10_H_12_] [217]. 

The phosphite, phosphinite, and thiophosphite derivatives of the *arachno*-decaborate anion [*arachno*-6,9-(R_2_R′P)_2_-B_10_H_12_] (R = R′ = OMe, OEt, OPh; R = Ph, R′ = OEt; R = OBu, R′ = Ph; R = R′ = SEt) were prepared by the direct reactions of decaborane(14) with the corresponding phosphorus compounds or via substitution of the Me_2_S and MeCN groups in [*arachno*-6,9-(Me_2_S)_2_-B_10_H_12_] and [*arachno*-6,9-(MeC≡N)_2_-B_10_H_12_], respectively [217,288,289,290].

The reaction of decaborane(14) with diphenylchlorophosphine in diethyl ether gives the 6,9-bis(chlorodiphenylphosphonium) derivative [*arachno*-6,9-(ClPh_2_P)_2_-B_10_H_12_], which, upon the treatment with dimethylamine in alcohols, lead to the corresponding phosphonites [6,9-(ROPh_2_P)_2_-B_10_H_12_] (R = Me, Et, CH_2_CH_2_OH) [290]. The reaction of [6,9-(ClPh_2_P)_2_-B_10_H_12_] with dimethylamine in aqueous solution water results in the bis(dimethylammonium) salt of the corresponding acid (Me_2_NH_2_)_2_[6,9-(OPh_2_P)_2_-B_10_H_12_] [290]. The 6,9-bis-(hydroxydiphenylphosphonium) derivative [6,9-(HOPh_2_P)_2_-B_10_H_12_] was prepared by the reaction of [6,9-(ClPh_2_P)_2_-B_10_H_12_] with water in acetone [290].

The 6,9-bis(chlorodiphenylphosphonium) derivative reacts with ammonia, hydrazine, primary aliphatic amines, and ethylenimine in alcohols to form the corresponding 6,9-bis(aminodiphenylphosphonium) derivatives [6,9-(R′RNPh_2_P)_2_-B_10_H_12_] (R = H, R′ =NH_2_, Me, Bu; R = R′ = CH_2_CH_2_) [290]. The reactions of decaborane(14) or [*arachno*-6,9-(R_2_S)_2_-B_10_H_12_] (R = Me, Et) with dimethylaminophosphines in refluxing benzene lead to the corresponding 6,9-bis(dimethylaminophosphonium) derivatives [6,9-(RR′(Me_2_N)P)_2_-B_10_H_12_] (R = R′ = NMe_2_; R = NMe_2_, R′ = Ph, Cl; R = R′ = Ph; R = Ph, R′ = Cl; RR′ = OCH_2_CH_2_O) [217]. 

The reaction of [6,9-(ClPh_2_P)_2_-B_10_H_12_] with NaN_3_ in ethanol results in the 6,9-bis-(azidodiphenylphosphonium) derivative [6,9-(N_3_Ph_2_P)_2_-B_10_H_12_] [290], which, upon the treatment with triphenylphosphine in refluxing benzene, gives the 6,9-bis(triphenylphosphineiminodiphenylphosphonium) derivative [6,9-(Ph_3_P=NPh_2_P)_2_-B_10_H_12_] [291].

The bifunctional derivatives [6,9-(XPh_2_P)_2_-B_10_H_12_] (X = Cl, OH, N_3_) were used for the synthesis of decaborane-based polymers [291,292]. The chemistry of decaborane-based polymers is considered in detail in the review [293]. The formation of decaborane-based polymers along with a small amount of [6,9-(dppf)_2_-B_10_H_12_] has also been reported in the reaction of decaborane(14) with 1,1′-bis(diphenylphosphino)ferrocene (dppf) [294].

**Figure 23:**
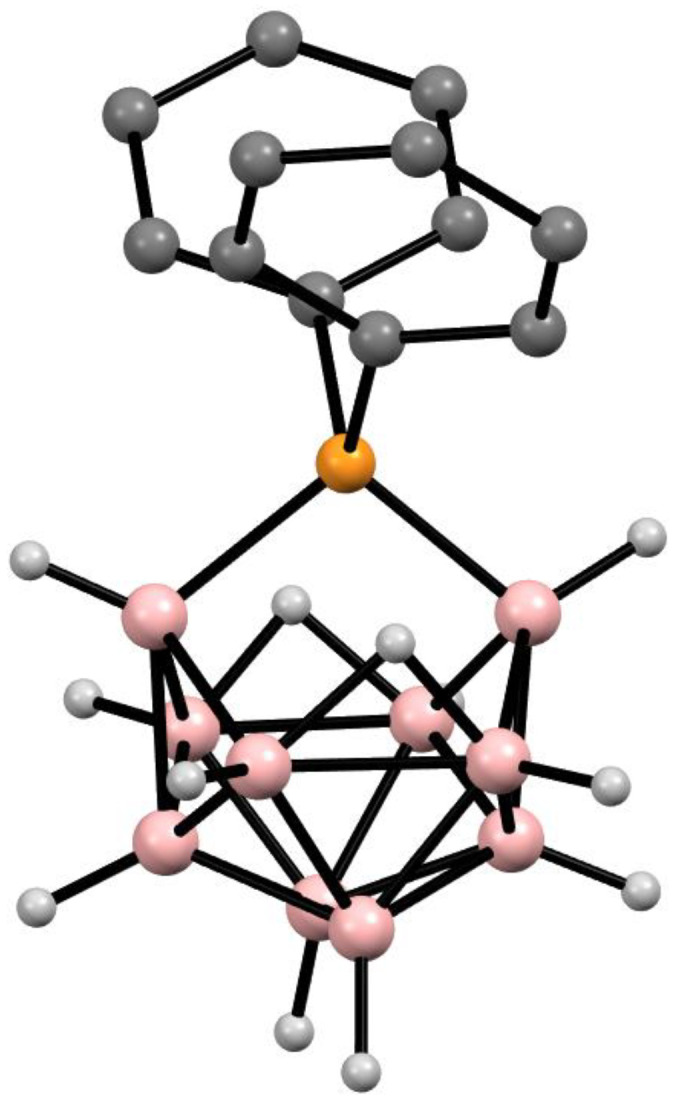
Solid state structure of the [*arachno*-μ-6,9-Ph_2_P-B_10_H_12_]^−^ anion. Hydrogen atoms of organic substituents are omitted for clarity.

The reaction of Na[B_10_H_13_] with tributylphosphine in acetonitrile in the presence of dry HCl leads to [*arachno*-6-Bu_3_P-9-MeC≡N-B_10_H_12_] [221]. The reaction of Na[B_10_H_13_] with diphenylchlorophosphine in diethyl ether leads to the diphenylphosphine derivative [*nido*-μ-5,6-Ph_2_P-B_10_H_13_] [295], whose structure was determined by single-crystal X-ray diffraction [296]. The same compound was reported to be formed in the reaction of the so-called “Grignard derivative” [B_10_H_13_MgI] formed by the treatment of decaborane(14) with MeMgI, with diphenylchlorophosphine in diethyl ether [297]. The diphenylphosphine derivative [*nido*-μ-5,6-Ph_2_P-B_10_H_13_] is easily deprotonated with triethylamine or sodium hydroxide to form the corresponding salts [295,297]. The solid state structure of the triphenylmethylphosponium salt (Ph_3_PMe)[*arachno*-μ-6,9-Ph_2_P-B_10_H_12_] was determined by single-crystal X-ray diffraction (Figure 23) [298].

It should be noted that the reactions of [6,9-(MeC≡N)_2_-B_10_H_12_] with low-coordinated phosphorus compounds, such as phosphaalkynes RC≡P (R = *t*-Bu, Ad), do not lead to substitution o hydrogens but to the incorporation of phosphorus into the decaborane basket with the formation of 11-vertex phosphoboranes [*nido*-RC(H)=PB_10_H_13_] [299,300].

## 8. Derivatives with a B-As Bond

The 6,9-bis(trialkyl/arylarsonium) derivatives [6,9-(R_3_As)_2_-B_10_H_12_] (R = Et, Ph) were prepared by the reactions of decaborane(14) or [6,9-(MeC≡N)_2_-B_10_H_12_] with the corresponding arsines in benzene or toluene [287]. The reaction of decaborane(14) with triethoxyarsine in benzene leads to [6,9-((EtO)_3_As)_2_-B_10_H_12_] [289]. 

## 9. Derivatives with a B-C Bond

Decaborane derivatives with a B-C bond are probably the most studied area of decaborane chemistry. Like the halogenation of decaborane, the direct alkylation reactions result in the substitution of hydrogen atoms at “the bottom” of the decaborane basket. The reaction of decaborane(14) with methyl bromide in carbon disulfide in the presence of AlCl_3_ at 80 °C gives a mixture of the 2-methyl [2-Me-B_10_H_13_], 1,2- and 3,4-dimethyl [1,2-Me_2_-B_10_H_12_] and [2,4-Me_2_-B_10_H_12_], 1,2,3- and 1,2,4-trimethyl [1,2,3-Me_3_-B_10_H_11_] and [1,2,4-Me_3_-B_10_H_11_], 1,2,3,4- and 1,2,3,5(or 8)-tetramethyl [1,2,3,4-Me_3_-B_10_H_10_], and [1,2,3,5(or 8)-Me_4_-B_10_H_10_] derivatives, which were chromatographically separated [301]. The methylation of decaborane(14) was also studied using methyl chloride [302,303]. 

**Figure 24:**
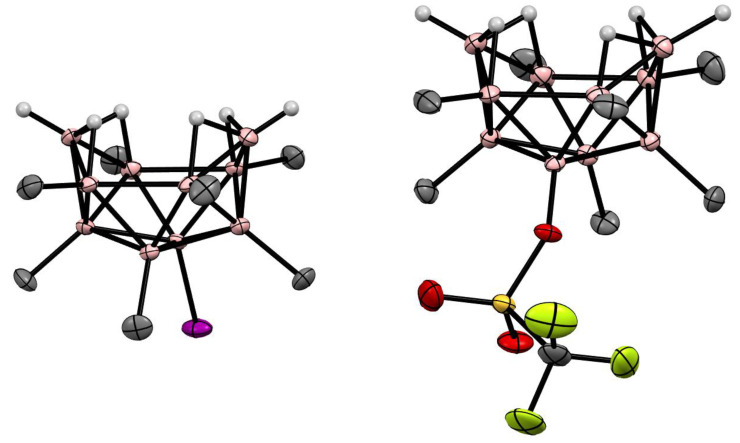
Solid state structures of [1-I-2,3,4,5,6,7,8-Me_7_-B_10_H_6_] (**left**) and [1-TfO-2,3,4,5,6,7,8-Me_7_-B_10_H_6_] (**right**). Hydrogen atoms of methyl groups are omitted for clarity.

The reaction of decaborane(14) with neat methyl iodide in the presence of AlCl_3_ at room temperature gives the 1,2,3,4-tetramethyl derivative [1,2,3,4-Me_4_-B_10_H_10_], whereas the reaction at 120 °C leads to the octasubstituted product [1-I-2,3,4,5,6,7,8-Me_7_-B_10_H_6_]. The similar octasubstituted derivative [1-TfO-2,3,4,5,6,7,8-Me_7_-B_10_H_6_] was obtained by the reaction of decaborane(14) with TfOMe in the presence of a catalytic amount of triflic acid at 120 °C. The solid state structures of [1-I-2,3,4,5,6,7,8-Me_7_-B_10_H_6_] and [1-TfO-2,3,4,5,6,7,8-Me_7_-B_10_H_6_] were determined by single-crystal X-ray diffraction (Figure 24) [304]. The methyl derivatives prepared can be easily deprotonated with a Proton Sponge to give the corresponding salts [304].

The reaction of decaborane(14) with ethyl bromide in carbon disulfide in the presence of AlCl_3_ under reflux gives a mixture of mono-, di-, and triethyl derivatives [305]. The monoethyl derivative of decaborane was prepared by the reaction of decaborane(14) with neat ethyl bromide in the presence of AlCl_3_ [306]. The solid state structure of the 1-ethyl derivative of decaborane [1-Et-B_10_H_13_] was determined by single-crystal X-ray diffraction [307].

The reaction of decaborane(14) with MeLi in benzene followed by treatment with HCl has been reported to give a mixture of the 6-methyl-, 6,5(or 8)- and 6,9-dimethyl derivatives of decaborane [308]. The reaction with EtLi in benzene gives the 6-ethyl derivative [6-Et-B_10_H_13_] [308]. The reaction of decaborane(14) with MeMgI has been shown to proceed by two routes. The major reaction yields the so-called “Grignard derivative” [B_10_H_13_MgI] and methane, and the minor reaction produces the 6-methyl derivative of decaborane. The reaction of the “Grignard derivative” with dimethyl sulfate produces a mixture of the 5- and 6-methyl derivatives of decaborane [309]. In a similar way, the reaction of decaborane(14) with EtMgI produces the “Grignard derivative” as the main product and the 6-ethyl derivative of decaborane as a by-product. The reaction of the “Grignard derivative” with [Et_3_O]BF_4_ or diethyl sulfate gives the 5-ethyl derivative of decaborane [5-Et-B_10_H_13_] [309]. A series of alkyl derivatives [R-B_10_H_13_] (R = butyl, amyl, hexyl, cyclohexyl, heptyl, octyl) was prepared by the reactions of the “Grignard derivative” with the corresponding alkyl fluorides [310]. The 6-benzyl derivative of decaborane [6-Bn-B_10_H_13_] can be prepared by the reaction of the “Grignard derivative” with benzyl chloride or Na[B_10_H_13_] with benzyl bromide [309,311,312]. 

Later, these reactions were re-examined, and it was shown that the first stage of the reaction of decaborane(14) with the alkyllithium reagents RLi is deprotonation of decaborane with the formation of Li[B_10_H_13_]. The reaction with the second equivalent of RLi produces Li_2_[*arachno*-6-R-B_10_H_13_], which, when treated with HCl, gives Li[*arachno*-6-R-B_10_H_14_] and then [*nido*-6-R-B_10_H_13_] (R = Me, *n*-Bu, *t*-Bu). The use of pre-prepared salts of the [B_10_H_13_]^−^ anion makes it possible to reduce the formation of by-products [237,313].

**Figure 25:**
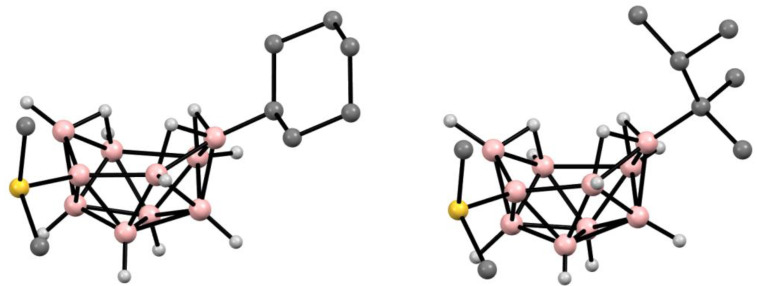
Solid state structures of [6-Chx-8-Me_2_S-B_10_H_11_] (**left**) and [6-Thx-8-Me_2_S-B_10_H_11_] (**right**). Hydrogen atoms of organic substituents are omitted for clarity.

**Figure 26:**
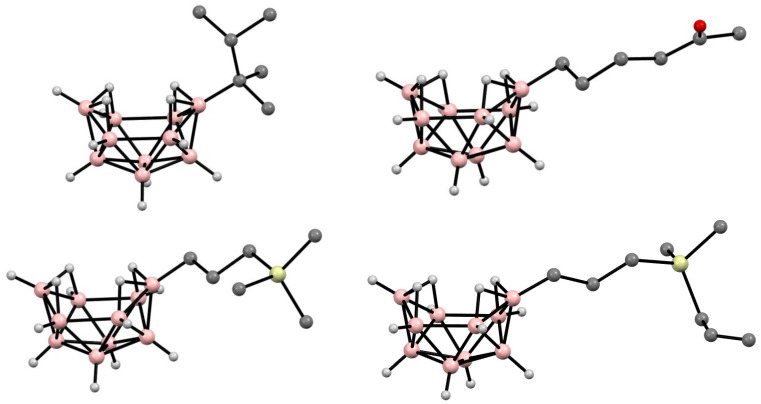
Solid state structures of [6-Thx-B_10_H_13_] (**top left**), [6-MeC(O)CH_2_CH_2_CH_2_CH_2_-B_10_H_13_] (**top right**), [6-Me_3_SiCH_2_CH_2_CH_2_-B_10_H_13_] (**bottom left**), and [6-H_2_C=CHCH_2_SiMe_2_CH_2_CH_2_CH_2_-B_10_H_13_] (**bottom right**). Hydrogen atoms of organic substituents are omitted for clarity.

Another approach to the 6-alkyl derivatives of decaborane includes the reactions of [*arachno*-6,9-(Me_2_S)_2_-B_10_H_12_] with alkenes in dichloromethane, resulting in [*nido*-6-R-8-Me_2_S-B_10_H_11_] (R = cyclohexyl, cyclohexenyl, hexyl, octyl, 2,3-dimethyl-1-butyl, 2,3-dimethyl-2-butyl, 2-methyl-2-butyl, (1R)-(+)-α-pinene, and (1S)-(−)-β-pinene), which can be reduced using Superhydride Li[Et_3_BH] in THF to [6-R-B_10_H_12_]^−^ and then protonated with HCl/Et_2_O to [6-R-B_10_H_13_] [222,237,313,314,315]. From the point of view of organic chemistry, these reactions can be considered as the hydroboration reactions. The solid state structures of [6-Chx-8-Me_2_S-B_10_H_11_] [316], [6-Thx-8-Me_2_S-B_10_H_11_] [237] (Figure 25), and [6-Thx-B_10_H_13_] [237] (Figure 26) were determined by single-crystal X-ray diffraction. 

Another convenient method for the synthesis of 6-alkyl derivatives of decaborane is based on the use of ionic liquids as a solvent. The reactions of decaborane(14) with terminal alkenes in biphasic ionic-liquid/toluene mixtures lead to the corresponding 6-alkyl derivatives [6-R-B_10_H_13_] (R = C_6_H_13_, C_8_H_17_, C_16_H_33_, CH(*i*-Pr)CH_2_CHMe_2_, (CH_2_)_2_C_6_H_5_, (CH_2_)_3_C_6_H_5_, (CH_2_)_6_Br, (CH_2_)_4_CH=CH_2_, (CH_2_)_6_CH=CH_2_, (CH_2_)_3_OC_3_H_7_, (CH_2_)_3_SiMe_3_, (CH_2_)_4_COMe, (CH_2_)_6_OAc, (CH_2_)_3_OBn, (CH_2_)_3_OH, and (CH_2_)_3_Bpin, norbornenyl) [317,318,319,320]. The best results were observed for reactions with [bmim]X (1-butyl-3-methylimidazolium, X = Cl^−^ or BF_4_^−^) and bmpyX (1-butyl-4-methylpyridinium, X = Cl^−^ or BF_4_^−^). The reaction mechanism includes the ionic-liquid-promoted formation of the [B_10_H_13_]^−^ anion, its addition to the alkene to form the [6-R-B_10_H_12_]^−^ anion, and, finally, the protonation of the last one to form the final product [6-R-B_10_H_13_] [314]. The solid state structures of [6-Me_3_Si(CH_2_)_3_-B_10_H_13_] and [6-MeC(O)(CH_2_)_4_-B_10_H_13_] were determined by single-crystal X-ray diffraction (Figure 26) [318].

**Figure 27:**
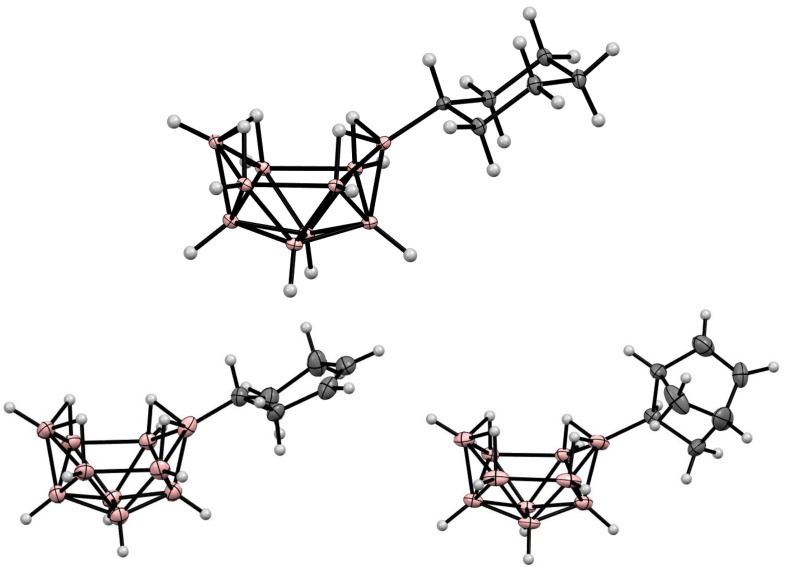
Solid state structures of [*nido*-6-C_6_H_11_-B_10_H_13_] (**top**), [6-(4′-cyclohexenyl)-B_10_H_13_] (**bottom left**) and [6-(5′-norbornenyl)-B_10_H_13_] (**bottom right**).

The 6-cyclohexyl derivative of decaborane [*nido*-6-C_6_H_11_-B_10_H_13_] was obtained in a low yield from the reaction of Cs_2_[*closo*-B_10_H_10_] with triflic acid in cyclohexane (Figure 27) [202,203]. In a similar way, the 6-hexyl derivative [*nido*-6-C_6_H_13_-B_10_H_13_] was isolated from the reaction of (NH_4_)_2_[*closo*-B_10_H_10_] with concentrated nitric acid in hexane [321].

The Cp_2_Ti(CO)_2_-catalyzed reactions of decaborane(14) with terminal alkenes have been found to result in the high-yield formation of 6-alkyl derivatives of decaborane [6-R-B_10_H_13_] (R = C_6_H_13_, C_8_H_17_, (CH_2_)_3_SiMe_3_) [322,323]. The reactions of decaborane(14) with equimolar amounts of bifunctional alkenes such as diallyldimethylsilane, 1,5-hexadiene, 1,4-cyclohexadiene, 1,5-cyclooctadiene, and 2,5-norbornadiene produce the corresponding decaborane derivatives with a double bond in the substituent [6-R-B_10_H_13_] (R = (CH_2_)_3_SiMe_2_CH_2_CH=CH_2_, (CH_2_)_4_CH=CH_2_, 4-cyclohexenyl, 5-cyclooctenyl, 5-norbornenyl) [322,323,324,325]. The solid state structures of [6-H_2_C=CHCH_2_SiMe_2_(CH_2_)_3_-B_10_H_13_] (Figure 26) [322], [6-(4′-cyclohexenyl)-B_10_H_13_] (Figure 27) [325], and [6-(5′-norbornenyl)-B_10_H_13_] (Figure 27) [324] were determined by single-crystal X-ray diffraction. 

**Figure 28:**
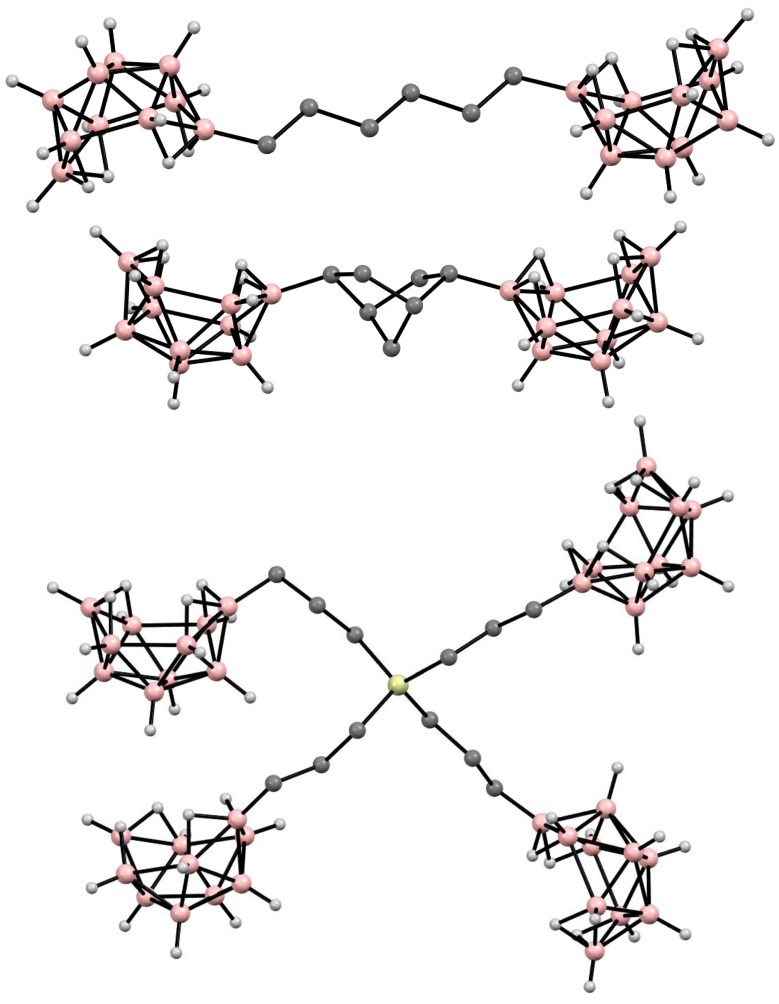
Solid state structures of [μ-6,6′-(CH_2_)_6_-(B_10_H_13_)_2_] (**top**), [μ-6,6′-(2″,5″-norbornyl)-(B_10_H_13_)_2_] (**middle**), and [μ^4^-6,6′,6″,6‴-Si-(6-(CH_2_)_3_-B_10_H_13_)_4_] (**bottom**). Hydrogen atoms of organic substituents are omitted for clarity.

The reactions of multifunctional alkenes with an excess amount of decaborane(14) produce the saturated linked-cage compounds with two ([μ-6,6′-Me_2_Si-(6-(CH_2_)_3_-B_10_H_13_)_2_], [μ-6,6′-(CH_2_)_6_-(B_10_H_13_)_2_], [μ-6,6′-(1″,5″-cyclooctyl)-(B_10_H_13_)_2_], and [μ-6,6′-(2″,5″-norbornyl)-(B_10_H_13_)_2_]) or four ([μ^4^-6,6′,6″,6‴-Si-(6-(CH_2_)_3_-B_10_H_13_)_4_]) decaborane units [322,323,325]. The solid state structures of [μ-6,6′-(CH_2_)_6_-(B_10_H_13_)_2_], [μ-6,6′-(2″,5″-norbornyl)-(B_10_H_13_)_2_], and [μ^4^-6,6′,6″,6‴-Si-(6-(CH_2_)_3_-B_10_H_13_)_4_] were determined by single-crystal X-ray diffraction (Figure 28) [322,323,325].

The derivatives with the two decaborane units [μ-6,6′-(1″,5″-cyclooctyl)-(B_10_H_13_)_2_] and [μ-6,6′-(2″,5″-norbornyl)-(B_10_H_13_)_2_] can also be prepared by the titanium-catalyzed reactions of decaborane(14) with [6-(5′-cyclooctenyl)-B_10_H_13_] and [6-(5′-norbornenyl)-B_10_H_13_], respectively [325]. 

The 6-alkyl derivatives of decaborane with substituents containing double bonds in the side chain (hexenyl, norbornenyl, etc.) are used for the synthesis of decaborane-based polymers and boron-containing ceramics [319,324,325,326,327,328,329,330,331,332].

**Figure 29:**
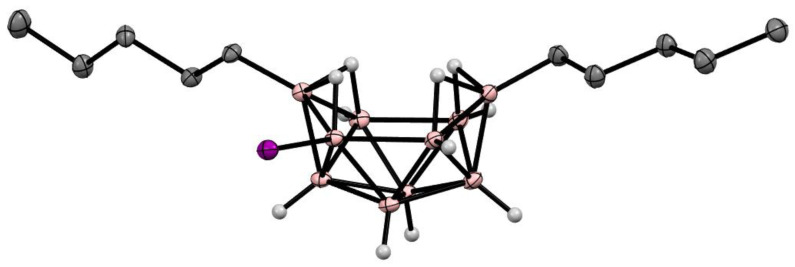
Solid state structure of [6,9-(C_5_H_11_)_2_-5-I-B_10_H_11_]. Hydrogen atoms of organic substituents are omitted for clarity.

The reactions of decaborane(14) with terminal alkenes in the presence of catalytic amounts of PtBr_2_ or H_2_PtCl_6_ lead to the 6,9-dialkyl derivatives *nido*-[6,9-R_2_-B_10_H_12_] (R = C_2_H_5_, C_3_H_7_, C_4_H_9_, C_5_H_11_) [333]. The reactions of [5-TfO-B_10_H_13_] and [5-I-B_10_H_13_] with 1-pentene in the presence of a catalytic amount of PtBr_2_ at 55 °C lead to the corresponding 6,9-dialkyl derivatives [6,9-(C_5_H_11_)_2_-5-TfO-B_10_H_11_] and [6,9-(C_5_H_11_)_2_-5-I-B_10_H_11_] [204]. The solid state structure of [6,9-(C_5_H_11_)_2_-5-I-B_10_H_11_] was determined by single-crystal X-ray diffraction (Figure 29) [204]. 

**Figure 30:**
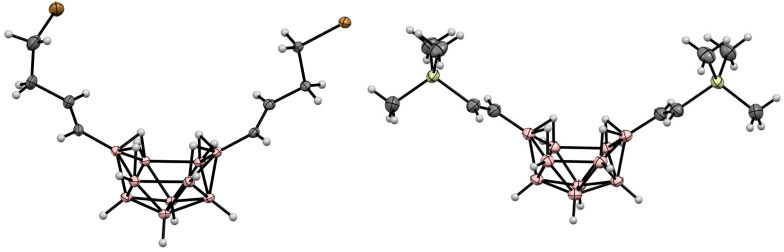
Solid state structures of [6,9-((*E*)-Br(CH_2_)_2_CH=CH)_2_-B_10_H_12_] (**left**) and [6,9-((*E*)-Me_3_SiCH=CH)_2_-B_10_H_12_] (**right**).

The reactions of decaborane(14) with terminal alkynes in toluene in the presence of [Cp*IrCl_2_]_2_ or [(*p*-cymene)RuCl_2_]_2_ as catalysts lead to the corresponding 6,9-di(β-alkenyl) derivatives of decaborane [6,9-((*E*)-RCH=CH)_2_-B_10_H_12_] (R = H, C_6_H_13_, C_6_H_5_, (CH_2_)_2_Br, (CH_2_)_3_Cl, SiMe_3_) [334,335]. The solid state structures of [6,9-((*E*)-Br(CH_2_)_2_CH=CH)_2_-B_10_H_12_] and [6,9-((*E*)-Me_3_SiCH=CH)_2_-B_10_H_12_] were determined by single-crystal X-ray diffraction (Figure 30) [334,335]. 

**Figure 31:**
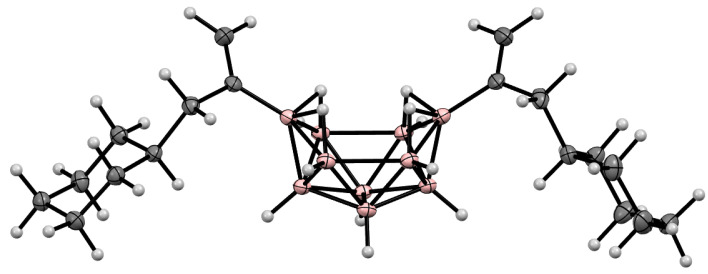
Solid state structure of [6,9-(*c*-C_6_H_11_CH_2_(H_2_C=)C)_2_-B_10_H_12_]. Hydrogen atoms of organic substituents are omitted for clarity.

In contrast to [(*p*-cymene)RuCl_2_]_2_, the reactions of decaborane(14) with terminal alkynes in the presence of [(*p*-cymene)RuI_2_]_2_ result in the 6,9-di(α-alkenyl) derivatives [6,9-(R(H_2_C=)C)_2_-B_10_H_12_] (R = C_6_H_13_, CH_2_-*c*-C_6_H_11_, (CH_2_)_2_Br, (CH_2_)_3_Cl) [334,335]. The solid state structure of [6,9-(*c*-C_6_H_11_CH_2_(H_2_C=)C)_2_-B_10_H_12_] was determined by single-crystal X-ray diffraction (Figure 31) [334,335]. 

**Figure 32:**
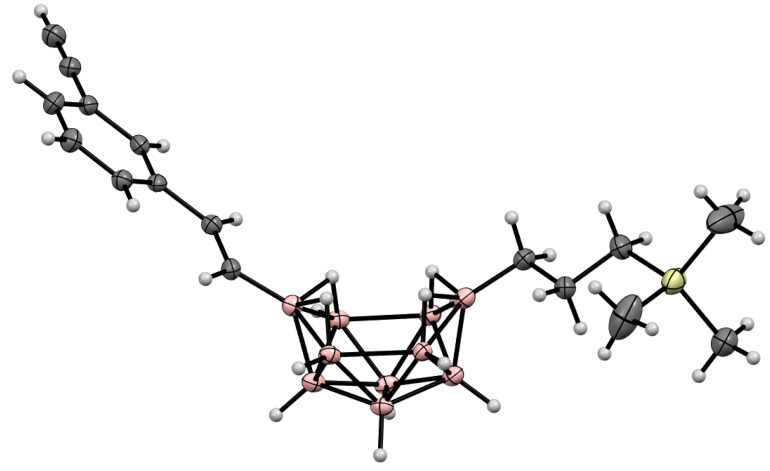
Solid state structure of [6-Me_3_Si(CH_2_)_3_-9-(*E*)-*m*-HC≡CC_6_H_4_CH=CH-B_10_H_12_].

In a similar way, the reactions of 6-alkyldecaboranes [6-R-B_10_H_13_] with terminal alkynes in the presence of [Cp*IrCl_2_]_2_ give asymmetrically substituted 6-alkyl-9-alkenyl-derivatives [6-R-9-((*E*)-R′CH=CH)_2_-B_10_H_12_] (R = (CH_2_)_3_SiMe_3_, R′ = H, C_6_H_5_, C_6_H_4_-*m*-CH≡CH; CH_2_CH=CH_2_; R = C_5_H_11_, R′ = H). The solid state structure of [6-Me_3_Si(CH_2_)_3_-9-(*E*)-*m*-HC≡CC_6_H_4_CH=CH-B_10_H_12_] was determined by single-crystal X-ray diffraction (Figure 32) [334,335]. 

**Figure 33:**
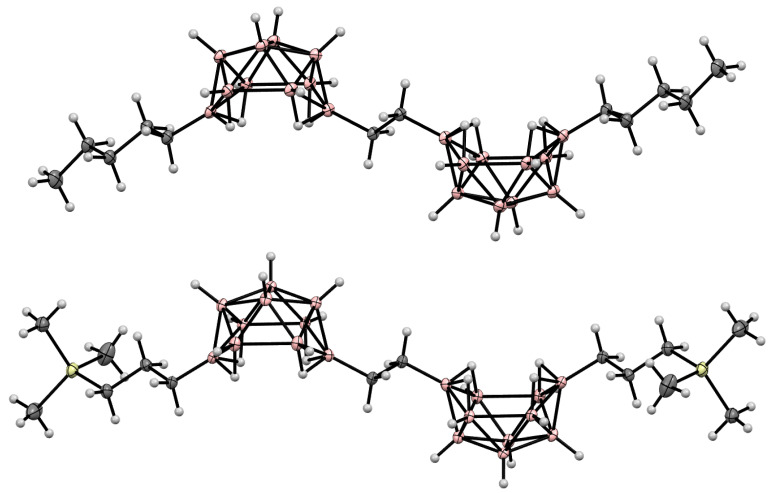
Solid state structures of [9,9′-μ-CH_2_CH_2_-(6-C_5_H_11_-B_10_H_12_)_2_] (**top**) and [9,9′-μ-CH_2_CH_2_-(6-Me_3_Si(CH_2_)_3_-B_10_H_12_)_2_] (**bottom**).

While [Cp*IrCl_2_]_2_ proved to be inactive for inducing the hydroboration of simple olefins, such as 1-pentene, by either decaborane or the 6-alkyl-decaboranes, it was found to catalyze the hydroboration of 6-alkyl-9-vinyldecaboranes [6-R-9-CH_2_=CH-B_10_H_12_] (R = C_5_H_11_, (CH_2_)_3_SiMe_3_) by 6-alkyl-decaboranes [6-R-B_10_H_13_] (R = C_5_H_11_, (CH_2_)_3_SiMe_3_) to yield linked-cage products [9,9′-μ-CH_2_CH_2_-(6-R-B_10_H_12_)_2_] (R = C_5_H_11_, (CH_2_)_3_SiMe_3_) (Figure 33) [335]. 

**Figure 34:**
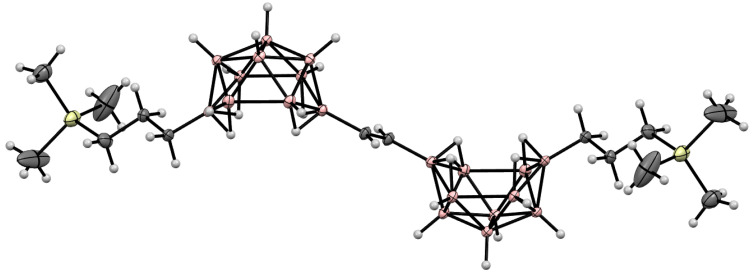
Solid state structure of [9,9′-μ-CH=CH-(6-Me_3_Si(CH_2_)_3_-B_10_H_12_)_2_].

The vinyl derivative [6-Me_3_Si(CH_2_)_3_-9-CH_2_=CH)_2_-B_10_H_12_] was found to readily undergo both homo- and cross-metathesis reactions in the presence of Grubbs’ II catalyst, giving the corresponding products [9,9′-μ-CH=CH-(6-Me_3_Si(CH_2_)_3_-B_10_H_12_)_2_] (Figure 34) and [6-Me_3_Si(CH_2_)_3_-9-RCH=CH-B_10_H_12_] (R = C_3_H_7_, (CH_2_)_4_Br, CH_2_SiMe_3_) [335]. 

**Figure 35:**
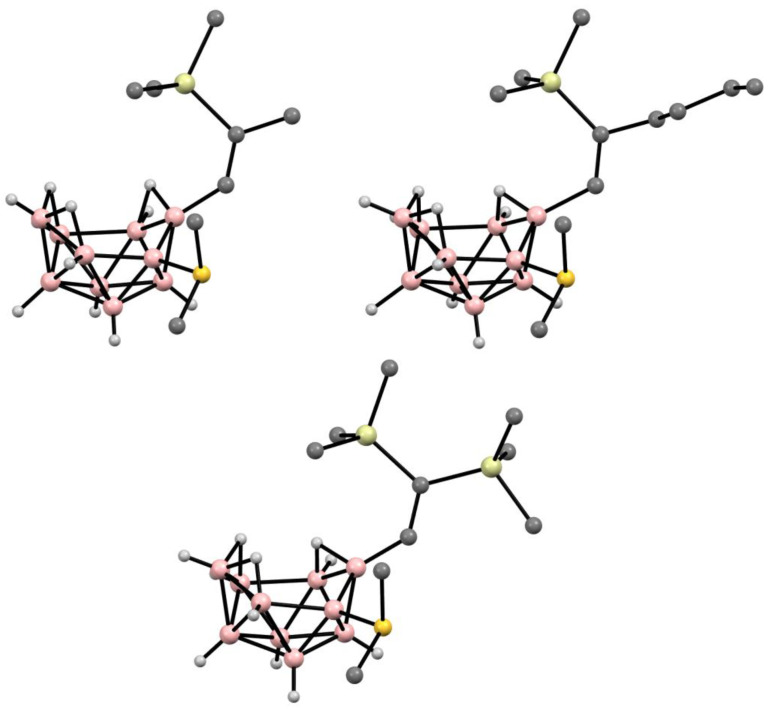
Solid state structures of [6-Me_3_Si(Me)C=CH-5-Me_2_S-B_10_H_11_] (**top left**), [6-Me_3_Si(Bu)C=CH-5-Me_2_S-B_10_H_11_] (**top right**), and [6-(Me_3_Si)_2_C=CH-5-Me_2_S-B_10_H_11_] (**bottom**). Hydrogen atoms of organic substituents are omitted for clarity.

Heating [*arachno*-6,9-(Me_2_S)_2_-B_10_H_12_] with silylated acetylenes Me_3_SiC≡CR (R = Me, Bu, SiMe_3_) leads to the corresponding trimethylsilyl alkenyl derivatives [*nido*-6-Me_3_Si(R)C=CH-5-Me_2_S-B_10_H_11_] [336,337]. The solid state structures of [6-Me_3_Si(Me)C=CH-5-Me_2_S-B_10_H_11_] [336], [6-Me_3_Si(Bu)C=CH-5-Me_2_S-B_10_H_11_] [337], and [6-(Me_3_Si)_2_C=CH-5-Me_2_S-B_10_H_11_] [337] were determined by single-crystal X-ray diffraction (Figure 35).

**Figure 36:**
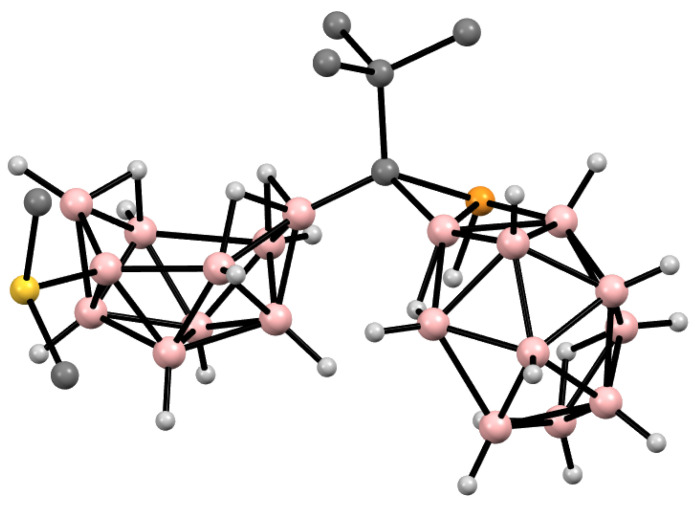
Solid state structure of [μ-6(*C*),6′(*C*),5′(*P*)-C(*t*-Bu)PH-(*nido*-8-Me_2_S-B_10_H_11_)(*nido*-B_10_H_12_)]. Hydrogen atoms of organic substituents are omitted for clarity.

The reaction of [6,9-(Me_2_S)_2_-B_10_H_12_] with the phosphaalkyne *t*-BuC≡P in refluxing benzene leads to [μ-6(*C*),6′(*C*),5′(*P*)-C(*t*-Bu)PH-(*nido*-8-Me_2_S-B_10_H_11_)(*nido*-B_10_H_12_)], in which two decaborane units are linked by the C(*t*-Bu)PH-bridge (Figure 36) [338]. 

**Figure 37:**
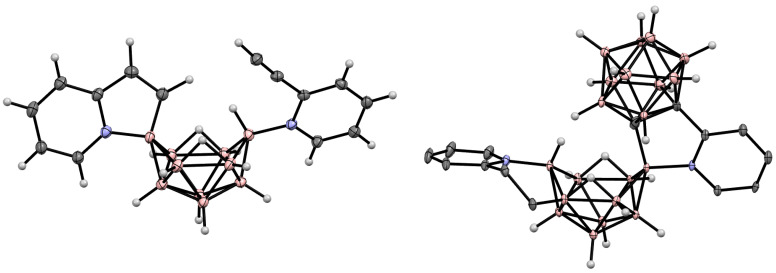
Solid state structures of [μ-(*exo*-6(*C*),*endo*-6(*N*)-CH=CH-*o*-C_5_H_4_N)-9(*N*)-HC≡C-*o*-C_5_H_4_N-*arachno*-B_10_H_11_] (**left**) and [μ-(*exo*-6(*C*),*endo*-6(*N*)-(*closo*-1′,2′-C_2_B_10_H_10_-2′-)-*o*-C_5_H_4_N)-μ-(*exo*-8(*C*),*exo*-9(*N*)-CH_2_CH_2_-*o*-C_5_H_4_N)-*arachno*-B_10_H_10_] (**right**). Hydrogen atoms of organic substituents in the left structure are omitted for clarity.

The derivatives [μ-(*exo*-6(*C*),*endo*-6(*N*)-CH=CH-*o*-C_5_H_4_N)-9(*N*)-HC≡C-*o*-C_5_H_4_N-*arachno*-B_10_H_11_] (Figure 34) [265] and [μ-(*exo*-6(*C*),*endo*-6(*N*)-(*closo*-1′,2′-C_2_B_10_H_10_-2′-)-*o*-C_5_H_4_N)-μ-(*exo*-8(*C*),*exo*-9(*N*)-CH_2_CH_2_-*o*-C_5_H_4_N)-*arachno*-B_10_H_10_] (Figure 37) [339] were isolated in minor amounts as products of the intramolecular hydroboronation of [*arachno*-6,9-(HC≡C-*o*-C_5_H_4_N)_2_-B_10_H_12_] during its thermolysis in 1,2-dichloroethane. In the latter compound, the formation of the *ortho*-carborane fragment occurs as a result of the reaction of the acetylene group of the substituent with the second molecule of the decaborane derivative. 

**Figure 38:**
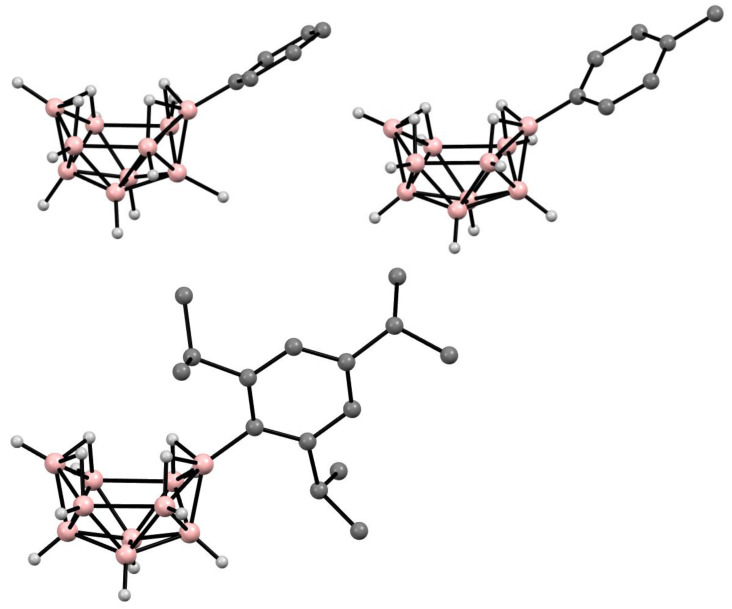
Solid state structures of [6-Ph-B_10_H_13_] (**top left**), [6-*p*-Tol-B_10_H_13_] (**top right**), and [6-(2′,4′,6′-*^i^*Pr_3_-C_6_H_2_-B_10_H_13_] (**bottom**). Hydrogen atoms of organic substituents are omitted for clarity.

The reactions of Cs_2_[*closo*-B_10_H_10_] with triflic acid or (NH_4_)_2_[*closo*-B_10_H_10_] with sulfuric acid in the presence of aromatic hydrocarbons produce the corresponding 6-aryl derivatives of decaborane [*nido*-6-Ar-B_10_H_13_] (Ar = Ph, C_6_H_4_-4-Me, C_6_H_3_-3,5-Me_2_, C_6_H_2_-2,4,6-Me_3_, C_6_H_2_-2,4,6-*^i^*Pr_3_, C_6_H_4_Cl, C_6_H_4_CF_3_) [202,203,321,340]. The solid state structures of [6-Ph-B_10_H_13_], [6-*p*-Tol-B_10_H_13_], and [6-(2′,4′,6′-*^i^*Pr_3_-C_6_H_2_-B_10_H_13_] were determined by single-crystal X-ray diffraction (Figure 38) [202,203,340].

The 6-phenyl derivative [6-Ph-B_10_H_13_] was also obtained by the reaction of decaborane(14) with PhLi, followed by the treatment with HCl in Et_2_O [237] as well as by the solid state pyrolysis of [*nido*-Ph_2_SnB_10_H_12_] at 95 °C [198].

**Figure 39:**
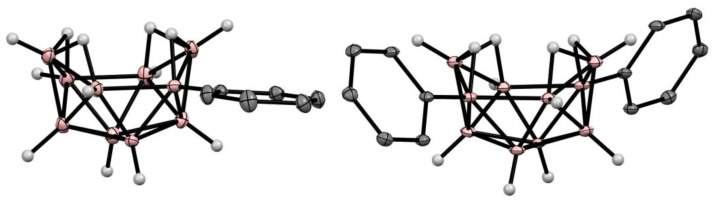
Solid state structures of [5-Ph-B_10_H_13_] (**left**) and [5,8-Ph_2_-B_10_H_12_] (**right**). Hydrogen atoms of organic substituents are omitted for clarity.

The pyrolysis of decaborane(14) in benzene at 200 °C gives the 5-phenyl derivative [5-Ph-B_10_H_13_] as the main product together with some amounts of the 6-isomer and 5,8-diphenyl derivative [5,8-Ph_2_-B_10_H_12_] [341]. The solid state structures of [5-Ph-B_10_H_13_] and [5,8-Ph_2_-B_10_H_12_] were determined by single-crystal X-ray diffraction (Figure 39) [341]. 

The pyrolysis of decaborane(14) in toluene at 250 °C affords the novel microporous polymer named “activated borane”, in which the decaborane clusters are interconnected by toluene moieties. Activated borane displays a high surface area of 774 m^2^ g^−1^, a thermal stability up to 1000 °C (under Ar), and a sorption capacity to emerging pollutants exceeding the capacity of commercial activated carbon [342].

**Figure 40:**
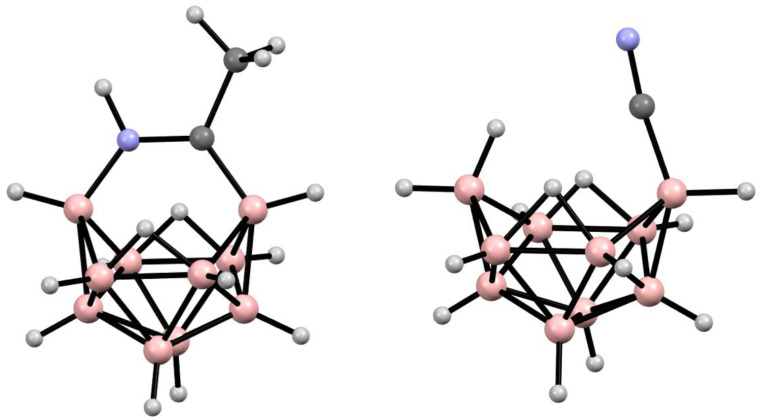
Solid state structures of the [*arachno*-μ-6(*C*),9(*N*)-MeC=NH-B_10_H_12_]^−^ (**left**) and [*arachno*-*endo*-6-N≡C-B_10_H_12_]^2−^ (**right**) anions.

Heating (HPS)[B_10_H_13_] in acetonitrile under reflux results in the formation of the bridged imino derivative (HPS)[*arachno*-μ-6(*C*),9(*N*)-MeC=NH-B_10_H_12_] (Figure 40) [343].

The reaction of decaborane(14) with sodium cyanide in water followed by the addition of CsCl gives Cs_2_[*arachno*-*endo*-6-N≡C-B_10_H_13_] [215]. The solid state structure of the trimethylphenylammonium salt (Me_3_NPh)_2_[*endo*-6-N≡C-B_10_H_13_] (Figure 40) [344] and lead complex {(Bipy)_2_Pb[*endo*-6-N≡C-B_10_H_13_]} [345] were determined by single-crystal X-ray diffraction. 

The reactions of decaborane(14) with sodium cyanide and dimethylsulfide or tetrahydrothiophene lead to the corresponding Na[*arachno*-6-N≡C-9-RR′S-B_10_H_12_] (R = R′ = Me, RR′ = (CH_2_)_4_). Na[6-N≡C-9-Me_2_S-B_10_H_12_] can also be prepared by the reaction of [6,9-(Me_2_S)_2_-B_10_H_12_] with sodium cyanide in dimethylsulfide [215].

## 10. Derivatives with an *exo*-Polyhedral B-B Bond

Decaborane derivatives with an *exo*-polyhedral B-B bond are rare, since the reaction of decaborane(14) with boron hydrides usually leads to the completion of the polyhedral backbone with the formation of tetradecahydro-*nido*-undecaborate [B_11_H_14_]^−^ [346] and dodecahydro-*closo*-dodecaborate [B_12_H_12_]^2−^ [3] anions and their derivatives. 

**Figure 41:**
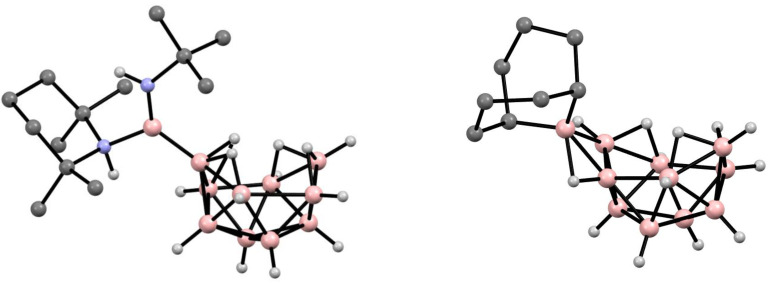
Solid state structures of [*nido*-6-(*t*-BuNH)(C_5_H_6_Me_4_NH)B-B_10_H_13_] (**left**) and [μ-5,6-(9-BBN)-B_10_H_13_] (**right**). Hydrogen atoms of alkyl groups are omitted for clarity.

The reactions of decaborane(14) with sterically hindered (alkyl/arylimino)(2,2,6,6-tetramethylpiperidino)boranes lead to the corresponding 6-substituted aminoborane derivatives [*nido*-6-(RNH)(C_5_H_6_Me_4_NH)B-B_10_H_13_] (R = *t*-Bu, C_6_H_3_-2,6-*^i^*Pr_2_) (Figure 41) [347].

The reaction of Na[B_10_H_13_] with 9-bora[3.3.1]bicyclononane (9-Br-BBN) in dichloromethane results in the formation of [μ-5,6-(9-BBN)-B_10_H_13_], where the 9-BBN group appears in the role of an asymmetric bridge between the B(5) and B(6) positions of the decaborane basket (Figure 41). It is noteworthy that upon the deprotonation of [μ-5,6-(9-BBN)-B_10_H_13_] with a Proton Sponge in dichloromethane, the 9-BBN bridging group migrates from the B(6) atom to the B(10) atom, which leads to the formation of an 11-vertex *nido*-structure (HPS)[μ-7,7-CH(CH_2_CH_2_CH_2_)_2_CH-B_11_H_12_], being a formal derivative of the [B_11_H_14_]^−^ anion [348].

Decaborane derivatives with an *exo*-polyhedral B-B bond also include isomeric *conjuncto*-decaboranes [B_10_H_13_]_2_, which consist of two *nido*-B_10_ units linked by a direct B-B bond. These compounds were first identified as trace impurities in technical decaborane (l4) [349]. In principle, there can be 11 different geometric isomers of *conjuncto*-decaborane [B_10_H_13_]_2_, 4 of which are in the form of enantiomeric pairs. Therefore, various routes (photolysis [350,351], pyrolysis [350,351], γ-irradiation [352], high-energy electron bombardment [351], silent electrical discharge [353]) for synthesizing these compounds have been developed. All of them, as a rule, lead to the formation of mixtures with different isomeric compositions. The solid state structures of the 1,1′- [353], 1,2′- [354], 1,5′- [352], 1,6′- [238], 2,2′- [355,356], and 2,6′- [356] isomers have been determined by single-crystal X-ray diffraction, whereas the 2,5′- [351], 5,5′- [351], and 6,6′- [357] isomers have been characterized by NMR spectroscopy.

**Figure 42:**
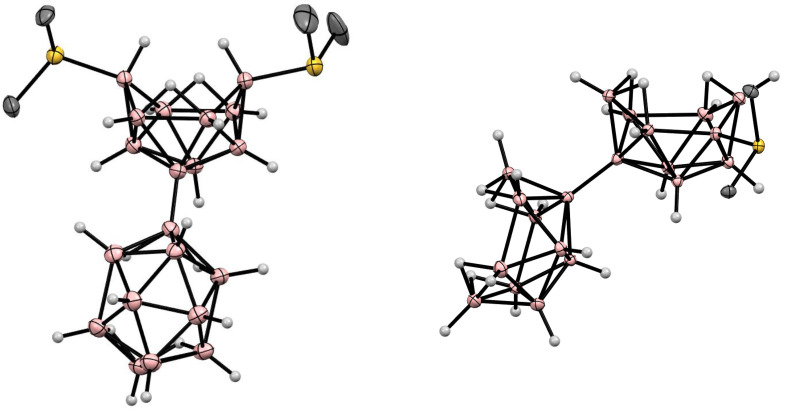
Solid state structures of [*arachno*-1-(6′-*nido*-B_10_H_13_)-6,9-(Me_2_S)_2_-B_10_H_11_] (**left**) and [*nido-*4-(2′-*nido*-B_10_H_13_)-5-Me_2_S-B_10_H_11_] (**right**). Hydrogen atoms of alkyl groups are omitted for clarity.

The asymmetric derivatives [*arachno*-1-(6′-*nido*-B_10_H_13_)-6,9-(Me_2_S)_2_-B_10_H_11_] and [*nido-*4-(2′-*nido*-B_10_H_13_)-5-Me_2_S-B_10_H_11_] (Figure 42) were isolated from the reaction of [1,5′-(*nido*-B_10_H_13_)_2_] with dimethylsulfide under reflux [238]. The first compound was also obtained by the thermolysis of [*arachn*o-6,9-(Me_2_S)_2_-B_10_H_12_] in refluxing toluene [238].

**Figure 43:**
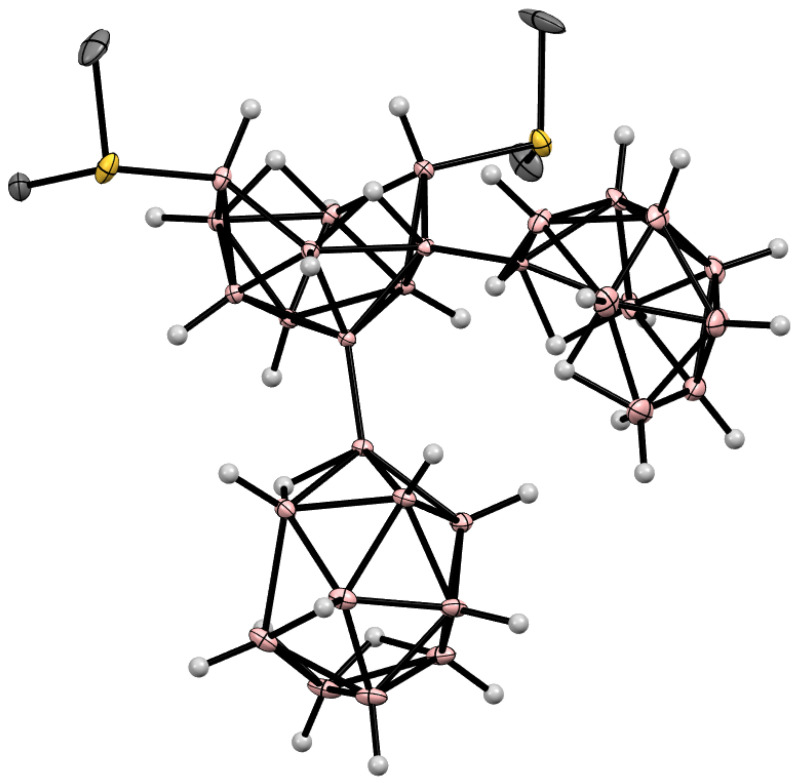
Solid state structure of [*arachno*-1,5-(6′-*nido*-B_10_H_13_)_2_-6,9-(Me_2_S)_2_-B_10_H_10_]. Hydrogen atoms of organic substituents are omitted for clarity.

The isomeric tridecaboranyl species [*arachno*-1,5-(6′-*nido*-B_10_H_13_)_2_-6,9-(Me_2_S)_2_-B_10_H_10_] (Figure 43) and [*arachno*-1,3-(6′-*nido*-B_10_H_13_)_2_-6,9-(Me_2_S)_2_-B_10_H_10_] were isolated from the products of the thermolysis of [*arachn*o-6,9-(Me_2_S)_2_-B_10_H_12_] in refluxing benzene [238,358].

**Figure 44:**
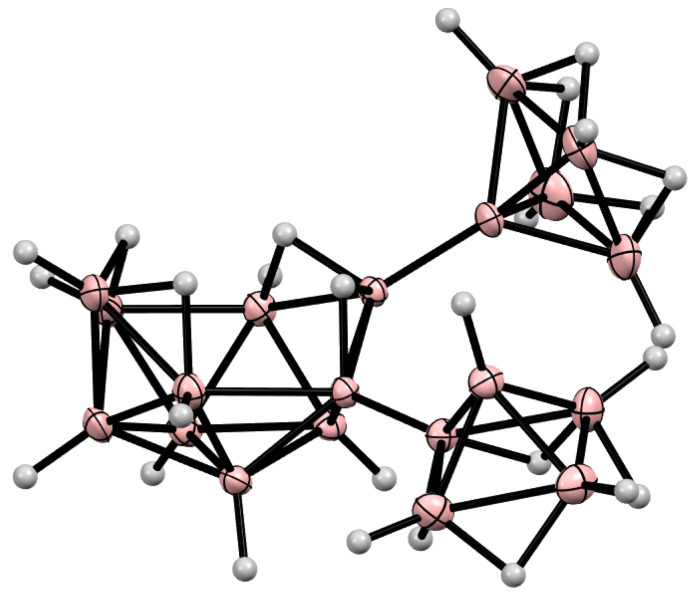
Structure of 5-(*nido*-pentaboran-2-yl)-6-(*nido*-pentaboran-1-yl)-*nido*-decaborane.

Here, it is also worth mentioning an unusual structure, in which two hydrogen atoms at positions 5 and 6 of the decaborane basket are replaced by pentaborane moieties—5-(*nido*-pentaboran-2-yl)-6-(*nido*-pentaboran-1-yl)-*nido*-decaborane, formed as a result of the long-term storage (23 years) of a sealed, under-vacuum pentaborane(9) sample under ambient lighting and temperature conditions (Figure 44) [339].

## 11. Decaborane-Related *conjucto*-Boranes [B_18_H_22_]

**Figure 45:**
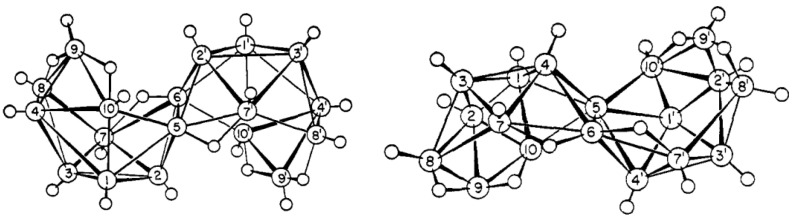
Structures and numbering of atoms in *syn-* (**left**) and *anti-* (**right**) isomers of [B_18_H_22_]. Reprinted with permission from Ref. [359]. Copyright (1968) the American Chemical Society.

Another type of compound worth considering here are the *conjuncto*-boranes [B_18_H_22_], in which two decaborane baskets are connected by a common edge. Octadecaborane(22) [B_18_H_22_] in the form of a mixture of *syn*- and *anti*-isomers (Figure 45) is formed on the hydrolysis of (H_3_O)_2_[*trans*-B_20_H_18_]·nH_2_O and can be separated by fractional crystallization [359]. More recently, a convenient method has been proposed for the synthesis of *anti*-[B_18_H_22_] by mild oxidation of (Me_4_N)[*nido*-B_9_H_12_] with iodine in toluene, which gives excellent yields (~80%) and thus provides a large-scale and safe route to this important polyborane cluster [360].

The photophysics of both isomers have been studied by UV–vis spectroscopic techniques and quantum chemical calculations. In an air-saturated hexane solution, *anti*-[B_18_H_22_] shows fluorescence with a high quantum yield, Φ_F_ = 0.97, and singlet oxygen O_2_(^1^Δ_g_) production (Φ_Δ_ ~ 0.008). Conversely, the isomer *syn*-[B_18_H_22_] shows no measurable fluorescence, instead displaying a much faster, picosecond nonradiative decay of excited singlet states [361]. Due to this, *anti*-[B_18_H_22_] can be considered as a potential blue laser material. The photophysical properties of *anti-*[B_18_H_22_] can be tuned by the partial substitution of hydrogen atoms with various functional groups. Because of this, combined with its high stability [362,363,364,365,366], *anti*-[B_18_H_22_] is attracting increasing research interest, while the *syn*-[B_18_H_22_] isomer has received much less attention.

**Figure 46:**
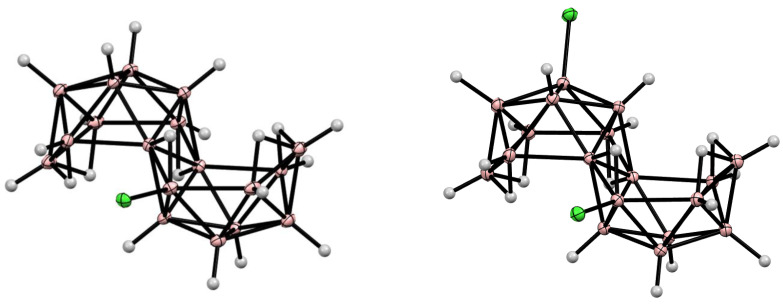

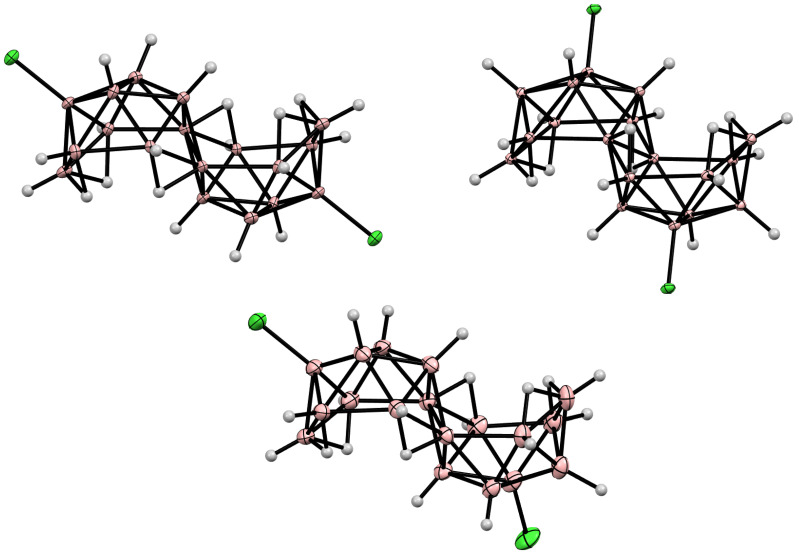
Solid state structures of *anti*-[7-Cl-B_18_H_21_] (**top left**), *anti*-[3,1′-Cl_2_-B_18_H_20_] (**top right**), *anti*-[4,4′-Cl_2_-B_18_H_20_] (**middle left**), *anti*-[3,3′-Cl_2_-B_18_H_20_] (**middle right**), and *anti*-[3,4′-Cl_2_-B_18_H_20_] (**bottom**).

The reaction of *anti-*[B_18_H_22_] with chlorine generated in situ from *N*-chlorosuccinimide (NCS) with HCl/dioxane in dichloromethane leads to the 7-chloro derivative *anti-*[7-Cl-B_18_H_21_] (Figure 46) [367]. The reaction of *anti-*[B_18_H_22_] with AlCl_3_ in tetrachloromethane results in a mixture of the 3,3′- and 3,4′-dichloro derivatives *anti-*[3,3′-Cl_2_-B_18_H_20_] (Figure 46) and *anti-*[3,4′-Cl_2_-B_18_H_20_] (Figure 45) together with minor amounts of the other isomeric dichloro derivatives *anti-*[4,4′-Cl_2_-B_18_H_20_] (Figure 46), *anti-*[3,1′-Cl_2_-B_18_H_20_] (Figure 46), and *anti-*[7,3′-Cl_2_-B_18_H_20_], as well as the 3- and 4-chloro derivatives *anti-*[3-Cl-B_18_H_21_] and *anti-*[4-Cl-B_18_H_21_] and the 3,4,3′- and 3,4,4′-trichloro derivatives *anti-*[3,4,3′-Cl_3_-B_18_H_19_] and *anti-*[3,4,4′-Cl_3_-B_18_H_19_], which were separated chromatographically [368]. 

**Figure 47:**
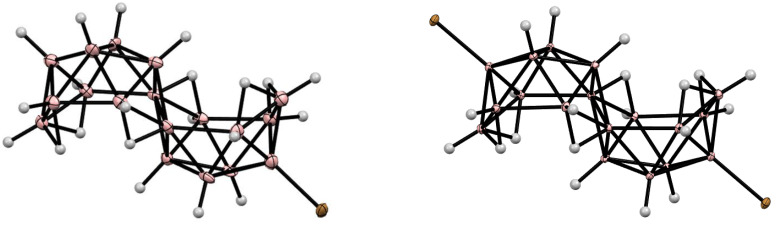
Solid state structures of *anti*-[4-Br-B_18_H_21_] (**left**) and *anti*-[4,4′-Br_2_-B_18_H_20_] (**right**).

The bromination of *anti*-[B_18_H_22_] with bromine in dichloromethane in the presence of AlCl_3_ leads to the 4-bromo or 4,4′-dibromo derivatives *anti*-[4-Br-B_18_H_21_] or *anti*-[4,4′-Br_2_-B_18_H_20_] depending on the reagent ratio (Figure 47) [369,370]. 

**Figure 48:**
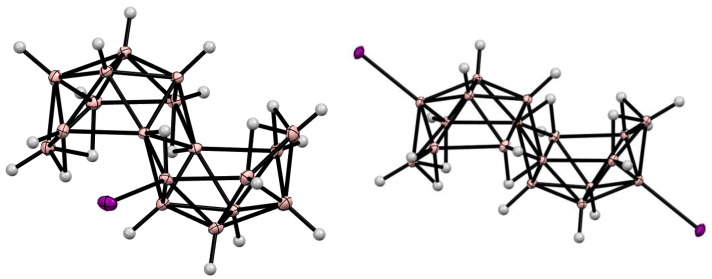
Solid state structures of *anti*-[7-I-B_18_H_21_] (**left**) and *anti*-[4,4′-I_2_-B_18_H_20_] (**right**).

The reaction of *anti*-[B_18_H_22_] with iodine in ethanol leads to the 4-iodo derivative *anti*-[7-I-B_18_H_21_] (Figure 48) [359,371], while the reaction with I_2_ or ICl in the presence of AlCl_3_ in dichloromethane results in the 4-iodo- and 4,4′-diiodo derivatives *anti*-[4-I_2_-B_18_H_21_] and *anti*-[4,4′-I_2_-B_18_H_20_] (Figure 48), depending on the reagent ratio [367,371].

The iodine atom in *anti*-[7-I-B_18_H_21_] can be substituted by various nucleophiles: the reaction with trifluoroacetamide in toluene in the presence of K_3_PO_4_ gives the corresponding *N*-boronated amide *anti*-[7-CF_3_CONH-B_18_H_21_]; the reactions with *t*-BuOK, 4-FC_6_H_4_OK, and 1-AdSK in toluene or tetrahydrofuran lead to the corresponding (thio)ethers *anti*-[7-RX-B_18_H_21_]. The reaction with potassium 2,6-dimethylthiophenolate in toluene results in the corresponding thioether *anti*-[7-(2′,6′-Me_2_C_6_H_3_S)-B_18_H_21_], while the reaction in tetrahydrofuran produces *anti*-[7-(2′,6′-Me_2_C_6_H_3_S(CH_2_)_4_O)-B_18_H_21_] [372]. The Pd-catalyzed reactions of *anti*-[7-I-B_18_H_21_] with CF_3_CONH_2_, *t*-BuOK, and 2,6-Me_2_C_6_H_3_OK in the presence of catalytic amounts of RuPhos Pd G4 and RuPhos in 1,4-dioxane lead to the corresponding derivatives with B-N and B-O bonds *anti*-[7-X-B_18_H_21_] [372].

**Figure 49:**
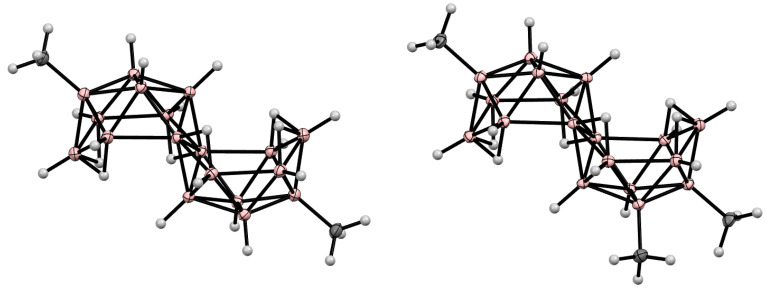

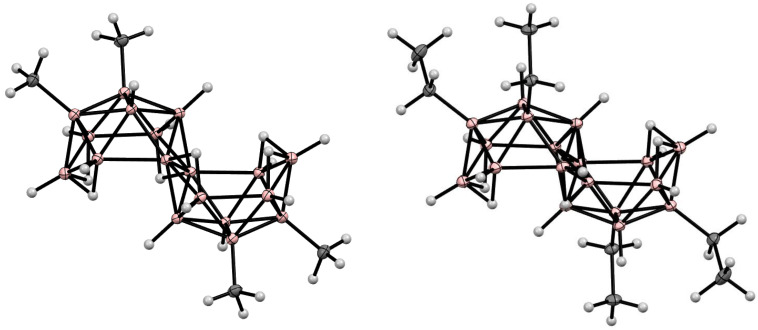
Solid state structures of *anti*-[4,4′-Me_2_-B_18_H_20_] (**top left**), *anti*-[3,4,4′-Me_3_-B_18_H_19_] (**top right**), *anti*-[3,3′,4,4′-Me_4_-B_18_H_18_] (**bottom left**), and *anti*-[3,3′,4,4′-Et_4_-B_18_H_18_] (**bottom right**).

The reaction of *anti*-[B_18_H_22_] with neat methyl iodide in the presence of AlCl_3_ at room temperature results in the 3,3′,4,4′-tetramethyl derivative *anti*-[3,3′,4,4′-Me_4_-B_18_H_18_] (Figure 49) as the main product, together with minor amounts of the 4,4′-dimethyl derivative *anti*-[4,4′-Me_2_-B_18_H_20_] (Figure 49), the 3,4,4′- and 3,3′,4-trimethyl derivatives *anti*-[3,4,4′-Me_3_-B_18_H_19_] (Figure 47) and *anti*-[3,3′,4-Me_3_-B_18_H_19_], as well as the 1,3,3′,4,4′- and 3,3′,4,4′,8-pentamethyl derivatives *anti*-[1,3,3′,4,4′-Me_5_-B_18_H_17_] and *anti*-[3,3′,4,4′,8-Me_5_-B_18_H_17_] and the 1,1′,3,3′,4,4′-hexamethyl derivative *anti*-[1,1′,3,3′,4,4′-Me_6_-B_18_H_16_] [373]. The similar reaction with ethyl iodide gives the 3,3′,4,4′-tetraethyl derivative *anti*-[3,3′,4,4′-Et_4_-B_18_H_18_] (Figure 49) [373].

**Figure 50:**
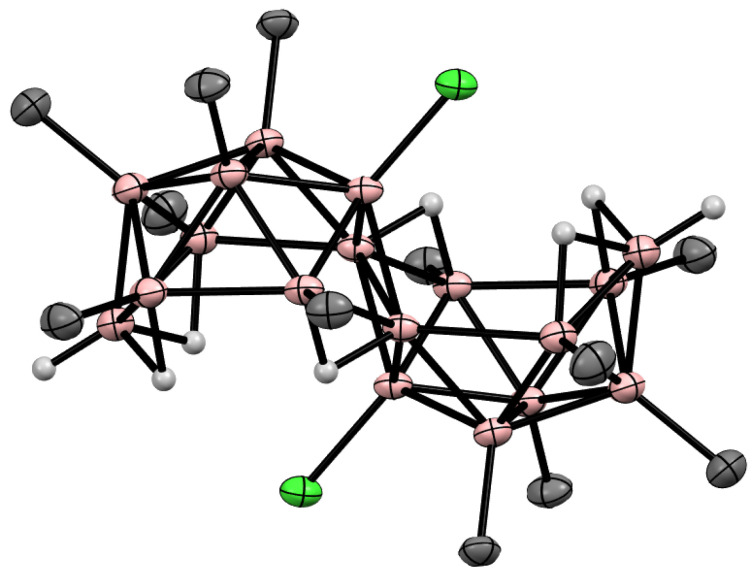
Solid state structure of *anti*-[2,2′-Cl_2_-1,1′,3,3′,4,4′,7,7′,8,8′,10,10′-Me_12_-B_18_H_8_]. Hydrogen atoms of organic substituents are omitted for clarity.

The dichloroundecamethyl *anti*-[2,2′-Cl_2_-1,1′,3,3′,4,4′,7,7′,8,8′,10′-Me_11_-B_18_H_9_], dichlorododecamethyl *anti*-[2,2′-Cl_2_-1,1′,3,3′,4,4′,7,7′,8,8′,10,10′-Me_12_-B_18_H_8_] (Figure 50), and dichlorotridecamethyl *anti*-[2,2′-Cl_2_-1,1′,3,3′,4,4′,7,7′,8,8′,9,10,10′-Me_13_-B_18_H_7_] derivatives were obtained by the reaction of *anti*-[B_18_H_22_] with methyl iodide in the presence of AlCl_3_ in dichloromethane at 55 °C [373,374].

**Figure 51:**
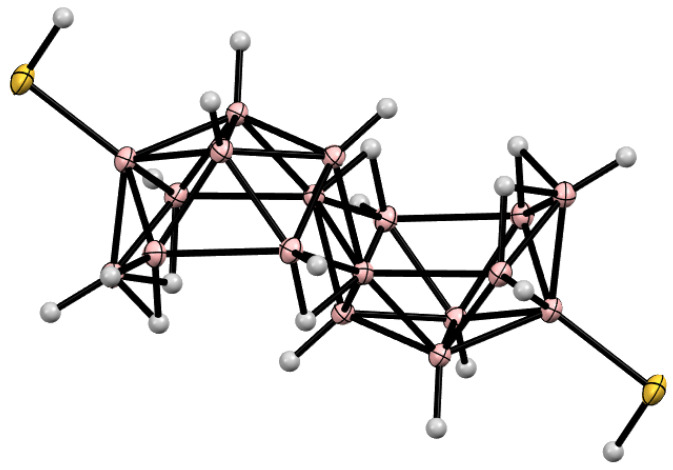
Solid state structure of *anti*-[4,4′-(HS)_2_-B_18_H_20_].

The reaction of *anti*-[B_18_H_22_] with elemental sulfur in the presence of AlCl_3_ at 125 °C leads to the 4,4′-dimercapto derivative *anti*-[4,4′-(HS)_2_-B_18_H_20_] (Figure 51) [375].

**Figure 52:**
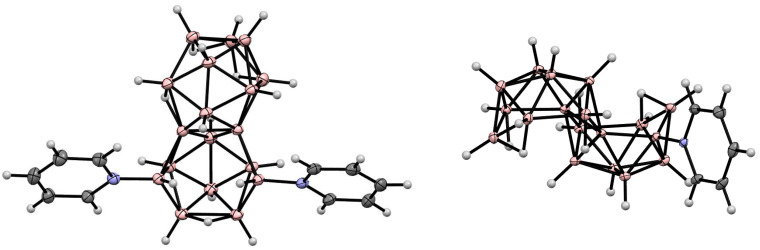
Solid state structures of *nido-arachno*-[6′,9′-Py_2_-B_18_H_20_] (**left**) and *anti*-[8-Py-B_18_H_21_] (**right**).

The reaction of *anti*-[B_18_H_22_] with pyridine in refluxing chloroform or benzene unexpectedly results in a two-fold substitution in one of the B_10_-baskets to form *nido-arachno*-[6′,9′-Py_2_-B_18_H_20_] (Figure 52) together with some amount of *anti*-[8′-Py-B_18_H_21_] (Figure 52) and [3′,8′-Py_2_-B_16_H_18_] as the main degradation product. In contrast to the thermochromic fluorescence of *nido-arachno*-[6′,9′-Py_2_-B_18_H_20_] (from 620 nm brick red at room temperature to 585 nm yellow at 8 K), *anti*-[8′-Py-B_18_H_21_] exhibits no luminescence [376,377]. The 6′,9′-disubstituted derivatives with 4-picoline [377], isoquinoline [378], and 5-hydroxyisoquinoline [379] were prepared in a similar way.

**Figure 53:**
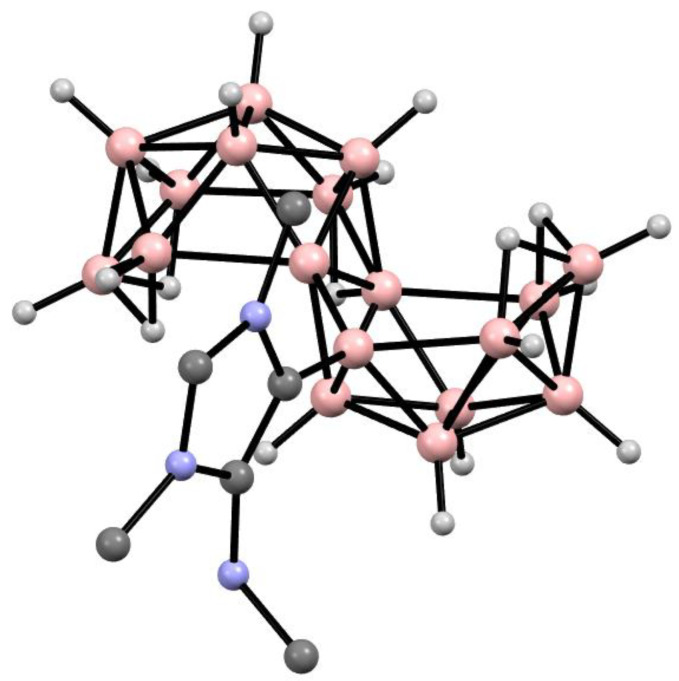
Solid state structure of *anti*-[7-{(MeNH)C_3_N_2_HMe_2_}-B_18_H_20_]. Hydrogen atoms of organic substituents are omitted for clarity.

The reaction of *anti*-[B_18_H_22_] with methyl isonitrile MeNC in benzene leads to *anti*-[7-{(MeNH)C_3_N_2_HMe_2_}-B_18_H_20_], in which a reductive trimerization of MeNC gives an unusual imidazole-based carbene, {(MeNH)C_3_N_2_HMe_2_}, that is stabilized by coordination to the macropolyhedral boron cluster (Figure 53) [380]. 

**Figure 54:**
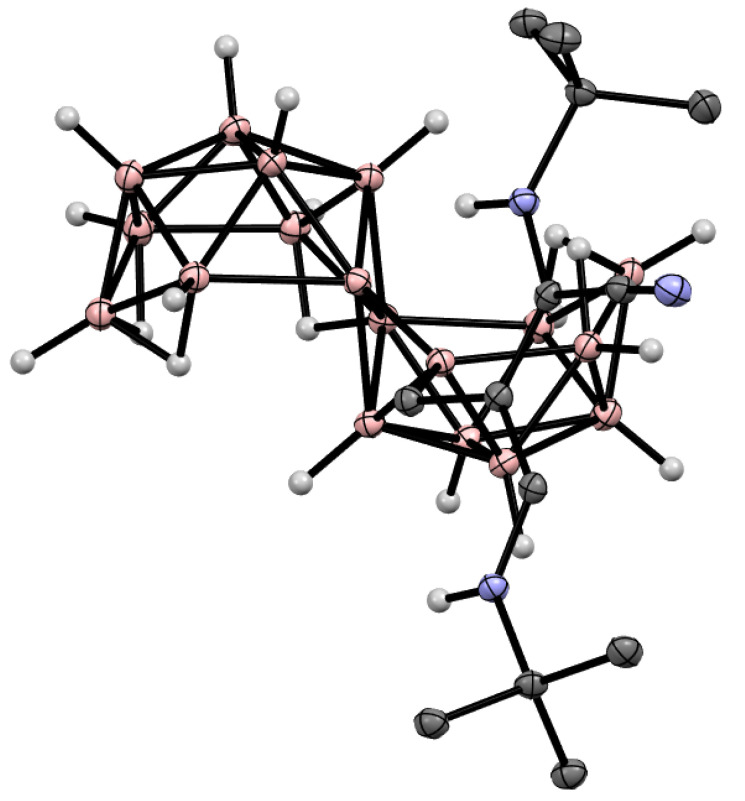
Solid state structure of *anti*-[7-{(*t*-BuNHCH){*t*-BuNHC(CN)}CH_2_}-B_18_H_20_]. Hydrogen atoms of organic substituents are omitted for clarity.

The reaction with *tert*-butyl isonitrile in 1,2-dichloroethane results in *anti*-[7-{(*t*-BuNHCH){*t*-BuNHC(CN)}CH_2_}-B_18_H_20_], in which a reductive oligomerization of *t*-BuNC has given the complex polynitrogen base {(*t*-BuNHCH){*t*-BuNHC(CN)}CH_2_:} formally as a zwitterionic carbene attached to the macropolyhedral boron cluster (Figure 54) [381].

**Figure 55:**
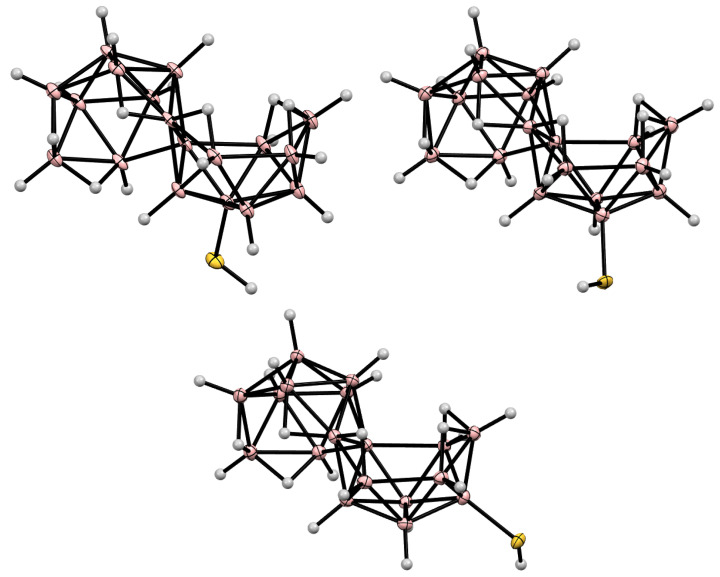
Solid state structures of *syn*-[1-HS-B_18_H_21_] (**top left**), *syn*-[3-HS-B_18_H_21_] (**top right**), and *syn*-[4-HS-B_18_H_21_] (**bottom**).

The synthesis of few substituted derivatives of *syn*-[B_18_H_22_] was reported. Heating *syn*-[B_18_H_22_] with sulfur in the presence of anhydrous AlCl_3_ at 125 °C results in a mixture of the isomeric mercapto derivatives *syn*-[1-HS-B_18_H_21_], *syn*-[3-HS-B_18_H_21_], and *syn*-[4-HS-B_18_H_21_], which were all separated by chromatography on silica (Figure 55) [382].

The 3- and 4-mercapto derivatives of *syn*-[B_18_H_22_], *syn*-[3-HS-B_18_H_21_], and *syn*-[4-HS-B_18_H_21_] were obtained as byproducts of thermolysis of nonathiaborane *arachno*-[SB_8_H_12_] in boiling cyclohexane [383]. The mercapto derivatives obtained are brightly luminescent under UV irradiation, making these compounds rare examples of a luminescent derivative of *syn*-[B_18_H_22_] [382,383].

**Figure 56:**
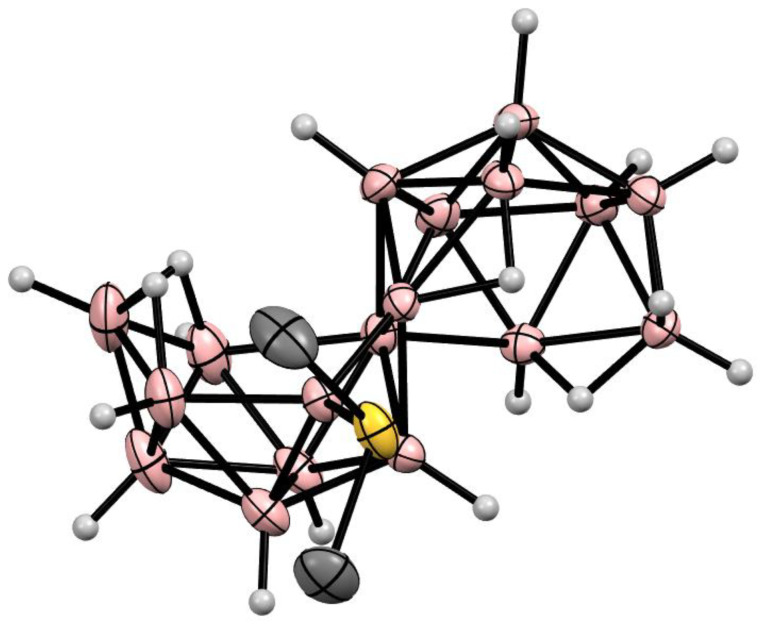
Solid state structure of *syn*-[7-Me_2_S-B_18_H_20_]. Hydrogen atoms of organic substituents are omitted for clarity.

The deprotonation of *syn*-[B_18_H_22_] with NaH in 1,2-dimethoxyethane, followed by the reaction with iodine and dimethylsulfide under reflux, results in the 7-dimethylsulfonium derivative *syn*-[7-Me_2_S-B_18_H_20_] (Figure 56) [384].

## 12. Conclusions

The purpose of this review was to give the most complete picture of the current state of the chemistry of decaborane and its derivatives. After its rapid development on the verge of the 1950s and 1960s, associated with the study of the chemistry of decaborane as a potential component of rocket fuels, the chemistry of decaborane was studied by many research groups. However, no comprehensive review elucidating it in full has appeared for more than 50 years, which certainly hindered the development of this important area of boron cluster chemistry. We would like to hope that this review will be useful both for young researchers just starting their way in boron chemistry and for researchers actively working in this field.

## Data Availability

No new data were created.
